# Genome Editing With TALEN, CRISPR-Cas9 and CRISPR-Cas12a in Combination With AAV6 Homology Donor Restores T Cell Function for XLP

**DOI:** 10.3389/fgeed.2022.828489

**Published:** 2022-05-23

**Authors:** Benjamin C. Houghton, Neelam Panchal, Simone A. Haas, Kay O. Chmielewski, Markus Hildenbeutel, Thomas Whittaker, Claudio Mussolino, Toni Cathomen, Adrian J Thrasher, Claire Booth

**Affiliations:** ^1^ Molecular and Cellular Immunology, UCL Great Ormond Street Institute of Child Health, London, United Kingdom; ^2^ Institute for Transfusion Medicine and Gene Therapy, Medical Center – University of Freiburg, Freiburg, Germany; ^3^ Center for Chronic Immunodeficiency, Faculty of Medicine, University of Freiburg, Freiburg, Germany; ^4^ Faculty of Biology, University of Freiburg, Freiburg, Germany

**Keywords:** TALEN, CRISPR (clustered regularly interspaced short palindromic repeat)/Cas9 (CRISPR associated protein 9)-mediated genome editing, Cas12a, X-linked lymphoproliferative disease (XLP), primary immunodefciencies, AAV6, T cell therapy, homology-directed repair

## Abstract

X-linked lymphoproliferative disease is a rare inherited immune disorder, caused by mutations or deletions in the *SH2D1A* gene that encodes an intracellular adapter protein SAP (Slam-associated protein). SAP is essential for mediating several key immune processes and the immune system - T cells in particular - are dysregulated in its absence. Patients present with a spectrum of clinical manifestations, including haemophagocytic lymphohistiocytosis (HLH), dysgammaglobulinemia, lymphoma and autoimmunity. Treatment options are limited, and patients rarely survive to adulthood without an allogeneic haematopoietic stem cell transplant (HSCT). However, this procedure can have poor outcomes in the mismatched donor setting or in the presence of active HLH, leaving an unmet clinical need. Autologous haematopoeitic stem cell or T cell therapy may offer alternative treatment options, removing the need to find a suitable donor for HSCT and any risk of alloreactivity. SAP has a tightly controlled expression profile that a conventional lentiviral gene delivery platform may not be able to fully replicate. A gene editing approach could preserve more of the endogenous regulatory elements that govern SAP expression, potentially providing a more optimum therapy. Here, we assessed the ability of TALEN, CRISPR-Cas9 and CRISPR-Cas12a nucleases to drive targeted insertion of *SAP* cDNA at the first exon of the *SH2D1A* locus using an adeno-associated virus serotype 6 (AAV6)-based vector containing the donor template. All nuclease platforms were capable of high efficiency gene editing, which was optimised using a serum-free AAV6 transduction protocol. We show that T cells from XLP patients corrected by gene editing tools have restored physiological levels of *SAP* gene expression and restore SAP-dependent immune functions, indicating a new therapeutic opportunity for XLP patients.

## Introduction

X linked lymphoproliferative disease (XLP) is a rare inherited immune system disorder, affecting 1–2:1,000,000 births (Booth et al., 2011). It arises due to mutations and deletions in the *SH2D1A* gene, which encodes an intracellular adaptor protein SLAM-associated protein (SAP) that is critical for relaying signals received at the cell surface by SLAM family receptors (Booth et al., 2011; Cannons et al., 2011). SAP is a small 128 amino acid cytoplasmic protein consisting of a single Src homology-2 (SH2) domain and a short C-terminal tail (Sayos et al., 1998). By binding to specific tyrosine-based motifs in the cytoplasmic tail of SLAM family receptors - such as SLAM, 2B4, NTB-A, Ly9 CD84 and CRACC - *via* an arginine residue in the SH2 domain (Cannons et al., 2011), SAP can recruit additional proteins that can activate downstream signaling cascades.

In the absence of SAP, several immune functions are affected, including reduced T cell and NK cell cytotoxicity, a lack of NKT cell development, defective CD4 T follicular helper (T_FH_) cell help to B cells leading to abnormal humoral function, and a reduced sensitivity to restimulation-induced cell death (RICD) that contributes to unconstrained immune responses to viral infection (Ma et al., 2006). These deficits give rise to a range of clinical manifestations, including haemophagocytic lymphohistiocytosis (HLH), dysgammaglobulinemia, lymphoma and autoimmunity (Panchal et al., 2018a).

Treatment options for XLP patients are limited and the only curative therapy is a haematopoietic stem cell transplant (HSCT), however, outcomes can be poor in the mismatched donor setting, as patients are at risk of graft-vs. host disease (GvHD). An autologous gene corrective approach could fulfil an unmet clinical need for patients lacking a well-matched donor. We have previously shown that lentiviral gene addition can restore SAP protein expression and immune function when delivered to haematopoietic stem cells (HSCs) and T cells, in several *in vitro* and *in vivo* models (Rivat et al., 2013; Panchal et al., 2018b). However, SAP has a tightly controlled expression profile, limited to T_convs_ (not T_reg_), NK and NKT cells. Within T cell subsets, SAP expression levels are upregulated after TCR engagement (Mehrle et al., 2005) and alter with memory or effector phenotypes (Hale et al., 2013), indicating an importance of finely tuned control and giving rise to concern that uncontrolled expression of this important signaling molecule in a conventional gene therapy procedure could cause further dysregulation. Although there is no direct evidence that irregular SAP expression is pathogenic, elevated (Geng et al., 2021) or decreased (Liu et al., 2021; Yang et al., 2021) levels are seen in several diseases, and SLAM signaling pathway alterations are implicated in autoimmunity (Comte et al., 2018; Dragovich and Mor, 2018; Malaer et al., 2019; Gartshteyn et al., 2021).

We hypothesised that a gene editing approach, using site-specific nucleases and a homology-directed repair (HDR) template to place a corrective SAP cDNA under the control of the full native promoter, could harness more of the endogenous regulatory elements that govern SAP expression, to potentially provide an optimal therapy. Genome editing is now a clinical reality due to the advent of highly site-specific and efficient DNA nucleases, including zinc-finger nucleases (ZFN), transcription activator–like effector-nucleases (TALENs) and CRISPR-Cas systems. All these platforms have entered the clinic, and the data from these trials is building an understanding of their safety profile and clinical efficacy (Tebas et al., 2014; Qasim et al., 2017; Frangoul et al., 2020; Stadtmauer et al., 2020; Gillmore et al., 2021).

TALENs are comprised of two customisable protein DNA binding domains, each expressed as a fusion protein to a *Fok*I endonuclease domain that upon dimerization creates a staggered DNA double stand break when the DNA binding domains are targeted to proximal loci on the DNA. Cas9 and Cas12a nucleases are guided by short RNAs that bind to their specific DNA loci *via* Watson-Crick base pairing. DNA cleavage is dependent on the presence of a protospacer adjacent motif (PAM) that takes the sequence NGG for *Streptococcus pyogenes* Cas9 (SpCas9) and TTTV for *Acidaminococcus* sp. Cas12a (AsCas12a). Cas9 is now the most widely used platform, creating a blunt end cut 3 nucleotides upstream of the PAM. However, Cas12a is an attractive novel editing platform, due to its creation of a 5bp overhang (downstream of PAM, from 18 nucleotides on the non-target strand, to 23 on the target strand) that may more efficiently stimulate HDR. The double strand break created by the nucleases can be harnessed to seamlessly insert therapeutic sequences by supplying a HDR template. For haematopoietic cells, delivery of this repair template *via* a genome of an adeno-associated virus with a serotype 6 (AAV6) capsid has shown the greatest efficiency, and capable of correcting immune dysfunctions in several primary immune deficiencies including SCID-X1, CD40L X-CGD and Wiskott Aldrich syndrome (Genovese et al., 2014; Kuo et al., 2018; Pavel-Dinu et al., 2019; Rai et al., 2020; Sweeney et al., 2021).

Most manifestations of XLP result from defects in T cell immunity. T cells offer an attractive therapeutic candidate, due to their accessibility and amenability to genome editing, and have an established safety record in many clinical trials for HIV and cancer immunotherapy (Tebas et al., 2014; Qasim et al., 2017; Panchal, et al., 2021). We have previously demonstrated that adoptive transfer of gene corrected T cells rescues a murine model of SAP deficiency alongside cellular and humoral abnormalities in XLP patient T cells (Panchal et al., 2018b). Here we set out to show proof of concept for gene editing platforms to correct T cell function using technologies which could then be transferred to HSC correction.

In order to allow treatment of the greatest number of patients, we designed a targeted integration of a *SAP* cDNA close to the initiation codon of the first exon. We identified TALEN, CRISPR-Cas9 and CRISPR-Cas12a nucleases, which were able to mediate high efficiency DNA cleavage in primary human T cells, with minimal off-target nuclease activity. When nuclease activity was coupled with an AAV6 HDR template, over 45% of XLP patient T cells showed targeted insertion of *SAP* cDNA. A serum-free transduction protocol optimised the editing procedure by reducing the AAV6 dose required. Finally, we show that gene edited XLP patient T cells have a restored, physiological level of SAP protein expression and restored SAP-dependent immune functions equal to that of healthy controls in immunoassays of T:B signaling, restimulation-induced cell death (RICD) and cytotoxicity. These data indicate that an autologous gene editing strategy could present a highly effective therapy for XLP patients.

## Results

### Targeting the *SH2D1A* Locus at High Efficiency in T Cells by TALEN, CRISPR-Cas9 and CRISPR-Cas12a

**FIGURE 1:**
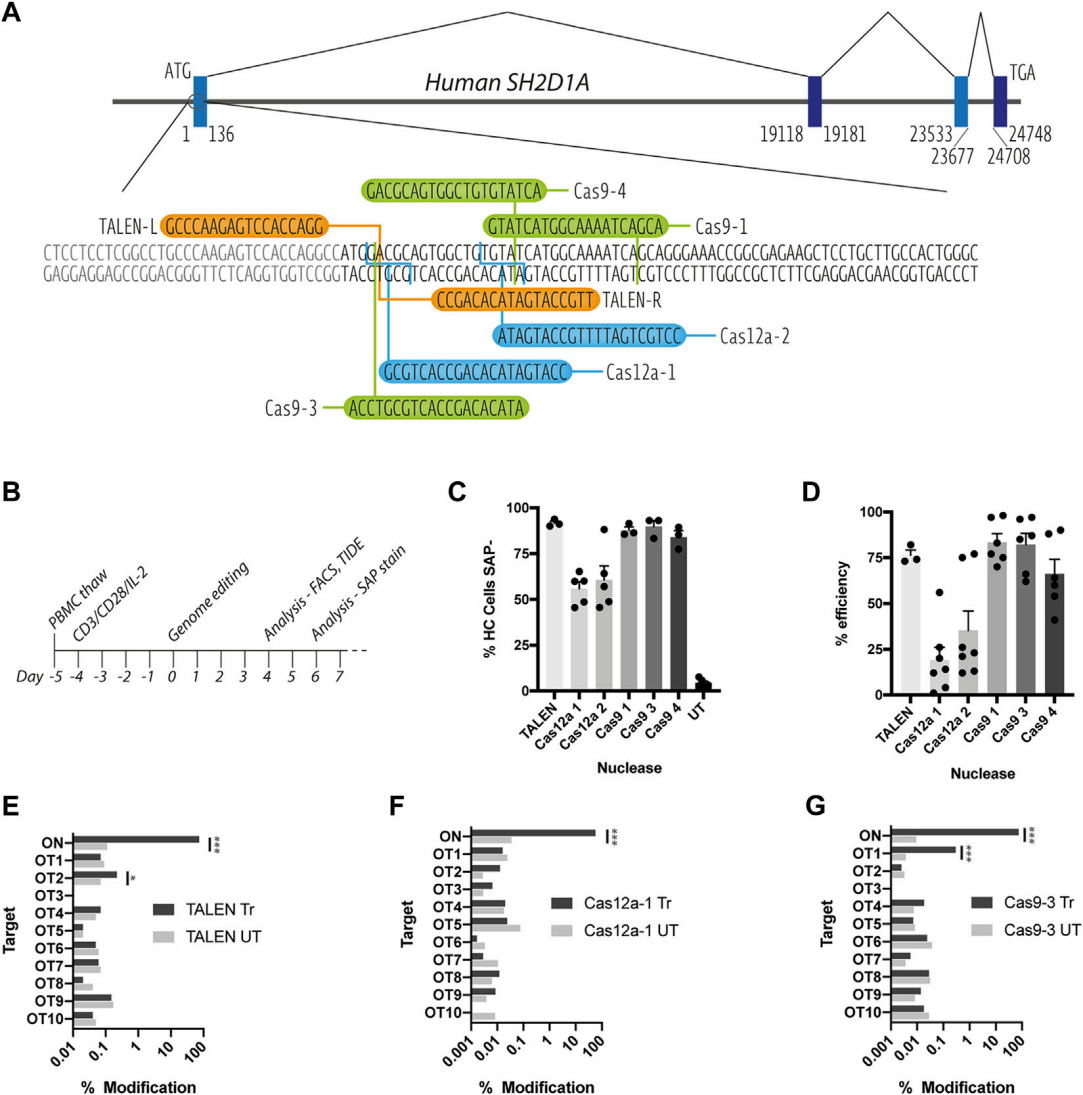
Assessment of on and off-target TALEN-, Cas9-and Cas12a-mediated activity. **(A)** Schematic diagram of the *SH2D1A* locus showing the start of the protein coding region of exon 1, TALEN-L and -R binding sites (orange), Cas9 (green) and Cas12a (blue) gRNA targets **(B)** T cell genome editing experimental timeline **(C)** Healthy control (HC) T cells nucleofected with TALEN mRNA, Cas9 or Cas12a RNPs, assessed for SAP expression by intracellular SAP staining and flow cytometry at d6 post nucleofection (n = 3–6, mean, SEM) **(D)** % INDEL frequency by TIDE analysis of sanger sequencing data of PCR amplicons amplified from nuclease treated T cell genomic DNA (n = 3–7, mean, SEM) **(E–G)** NGS-generated data of modifications at on target (ON) and *in silico* predicted off-target loci (OT1-10) for [E] TALEN [F] Cas12a-1 and [G] Cas9-3 nucleases (n = 1, treated (Tr) vs. untreated (UT), OT sites marked *p < 0.0001).

To determine the feasibility of a gene correction strategy for XLP, we firstly aimed to determine the optimal nuclease platform for creating the site-specific DNA double strand break. A TALEN pair, three S. p.Cas9 guide RNAs (gRNA) and two A.s.Cas12a crRNAs were identified targeting loci early in exon1 of the *SH2D1*A gene (Figure 1A). Stimulated PBMCs were nucleofected with either *in vitro*-transcribed TALEN mRNA, or ribonucleoprotein (RNP) complexes of Cas9 (Alt-R^®^ S. p.HiFi Cas9 V3, IDT) or Cas12a (Alt-R^®^ AsCas12a Ultra, IDT) protein and their respective guides, according to the timeline (Figure 1B).

All three nuclease platforms mediated efficient gene editing with TALEN and CRISPR-Cas9 nucleofection resulting in >90%, and CRISPR-Cas12a >50% knock down of SAP protein, when assessed using intracellular staining and flow cytometry (Figure 1C and Supplementary Figure S1). This data was supported by TIDE analysis detection of insertions and deletions (INDELs) created by non-homologous end-joining DNA repair of DNA double strand breaks (Figure 1D). TALEN, Cas12a-1 and Cas9-3 were selected for further testing due their proximity to the start codon of the *SAP* coding sequence.

### Minimal Detection of off Target Nuclease Activity Across Nuclease Platforms

To assess on and off-target cutting at more depth, we performed targeted next-generation sequencing (NGS) at the on-target (ON) locus and the top 10 *in silico* predicted off-target sites (OT1-10) for TALENs, Cas12a-1 and Cas9-3 nucleases (Determined by PROGNOS and Benchling online software, respectively, see Supplementary Table S1). NGS confirmed high efficiency modification at the on-target locus across all platforms giving a modification rate of 74, 57, and 75% for TALEN, Cas12a-1 and Cas9-3 respectively (Figures 1E–G). We detected off-target activity at two intronic loci (TALEN OT2 and Cas9-3 OT1) at low frequency (0.22 and 0.29% respectively) (Figures 1E–G). TALEN OT2 is in the third intron of the *TET1* (Ten-eleven translocation methylcytosine dioxygenase 1) gene while Cas9-3 OT1 occurs in the 24th intron of *RPTOR* (Regulatory-associated protein of mTOR). In both cases, the distance from the corresponding exon boundaries (up/downstream) are 21900/43664bp and 451/14567bp away, respectively (Supplementary Figure S2).

### *SH2D1A*-Targeted DNA Breaks can be Harnessed for HDR-Driven Insertion of *SAP* cDNA

**FIGURE 2:**
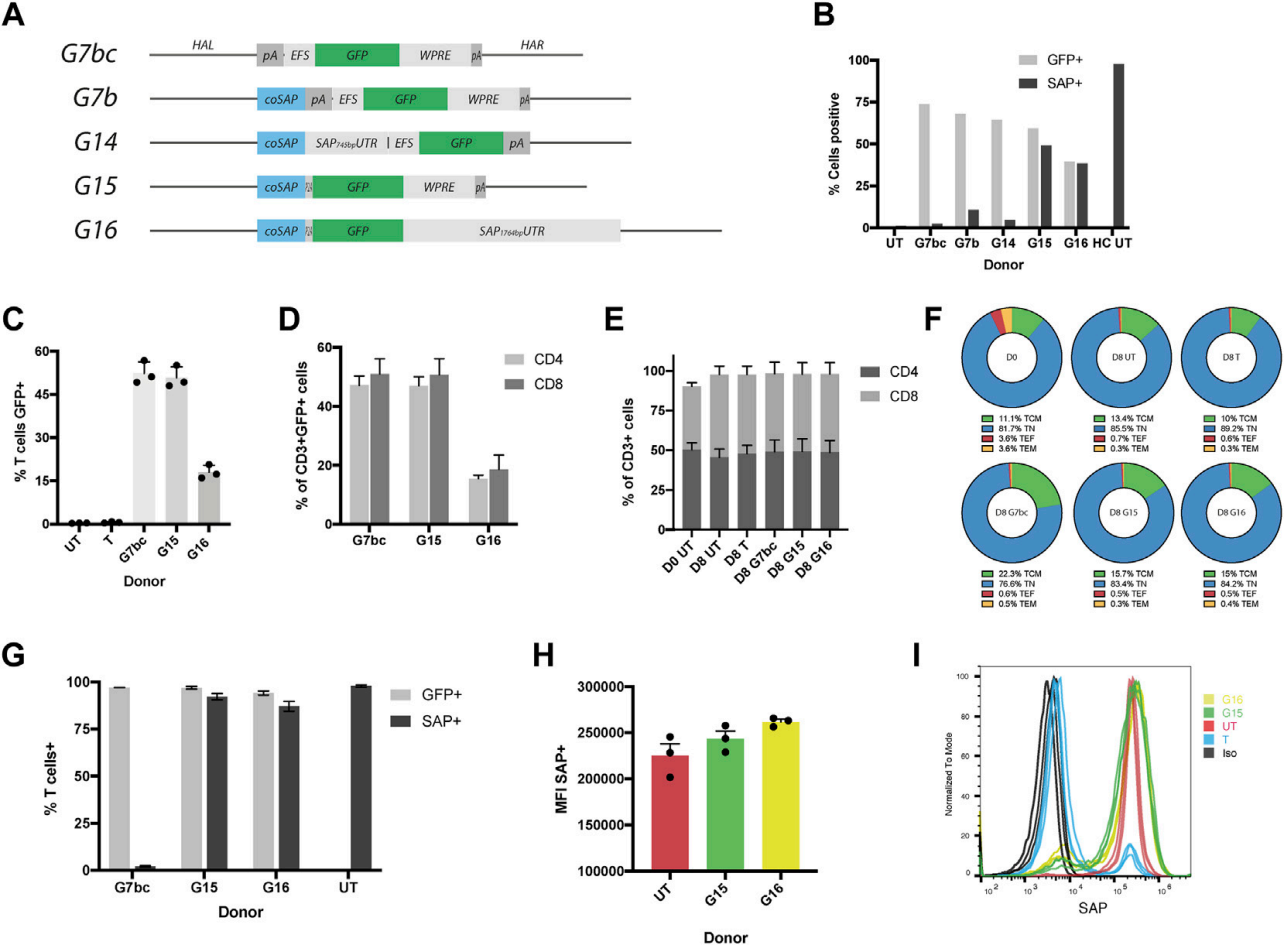
HDR donors mediate efficient integration and drive physiological levels of SAP expression. **(A)** Schematic diagrams of HDR donors designed for targeted insertion at the *SH2D1A* locus: G7bc (Homology arm left (HAL)-bovine growth hormone poly adenylation signal (bGHpA), EF1aShort (EFS), enhanced green fluorescent protein (GFP), woodchuck post-transcriptional regulatory element (WPRE), SV40pA, homology arm right (HAR)); G7b (HAL, codon optimised SAP cDNA (coSAP), bGHpA, EFS, GFP, WPRE, SV40pA, HAR); G14 (HAL, coSAP, 745bp SAP proximal 3′untranslated region (SAP_745bp_UTR), EFS, GFP, WPRE, SV40pA, HAR); G15 (HAL, coSAP, P2A, GFP, WPRE, SV40pA, HAR); G16 (HAL, coSAP, P2A, GFP, 1764bp proximal 3′untranslated region (SAP_1764bp_UTR), HAR). All constructs are flanked at the 5 and 3′ by inverted terminal repeats (ITRs) of an AAV2 viral genome (not shown). **(B)** Stimulated XLP patient T cells nucleofected with TALEN mRNA and transduced with HDR donors G7bc, G7b, G14, G15 and G16 alongside untreated patient (UT) and healthy control cells (HC), graph shows %GFP+ (unstained cells) and %SAP+ (intracellularly stained cells), analysed by flow cytometry (n = 1) **(C–I)** Healthy control T cells edited with TALEN and HDR donors, FACS-sorted on GFP + for G/H, n = 3, mean, SEM throughout) **(C)** % T cells positive for GFP (mean: G7bc, 52%; G15,50%; G16,18%) compared to untreated (UT) and TALEN-only (T) controls. **(D)** % of CD3+GFP + T cells expressing either CD4 or CD8 on cell surface **(E)** % CD4^+^ and CD8^+^ in CD3^+^ T cell gate at day 0 (D0) and day 8 (D8 = 4 days post edit). **(F)** CD62L/CD45RA T cell memory phenotype staining in CD4^+^ cell subset: T central memory (TCM) = CD62L + CD45RA-, Naïve (TN) = CD62L + CD45RA+, T effector (TF) = CD62L-CD45RA-,T effector memory (TEM) = CD62L-CD45RA- **(G)** Post-FACS-sorting %GFP+ and %SAP+ in healthy control T cells edited with G7bc, G15 or G16 HDR donors analysed by flow cytometry on unstained cells and intracellularly SAP stained cells respectively **(H)** Mean fluorescent intensity (MFI) of SAP + cells compared to untreated healthy control (UT) **(I)** Representative flow histogram shows comparable MFI between gene edited (G15, G16) and untreated healthy control cells. Cells treated with TALEN only (T) have both SAP+ and SAP- population, the latter overlaying the isotype control (Iso).

To determine if we could harness the HDR pathway to insert a corrective *SAP* cDNA under its native promoter, we designed a series of donor templates for delivery *via* AAV6 vector (Figure 2A). Donors were constructed into an AAV vector genome using Gibson assembly, to maintain the transition from promoter regions into a codon optimised *SAP* cDNA without restriction enzyme sequences. To allow for simplified identification of positively recombined clones, a *GFP* reporter gene was included, either as a separate expression cassette (G7b, G14), or co-expressed with SAP and cleaved during translation *via* a P2A self-cleaving peptide (G15, G16). In addition, we wanted to compare the endogenous *SH2D1A* 3′untranslated region (UTR) to a woodchuck post-transcriptional element (WPRE). We therefore cloned either the full annotated UTR (1764bp, G16) or a shorter 745bp fragment that contains all the validated microRNA binding sites for the SAP3′UTR, and fills the AAV packaging capacity of the two-cassette format (G14) (Figure 2A). A *GFP*-only donor was constructed as a negative control, alongside giving the option to create a functional knockout of *SAP* in healthy control cells, which could provide an additional *in vitro* model (G7bc).

Donor constructs were tested by transducing stimulated XLP patient-derived T cells 15 min post-electroporation of TALEN mRNA (1 × 10^5^ GC/cell). HDR efficiency was assessed by measuring the levels of GFP and SAP protein expression at day 5 post procedure. All donors were able to mediate HDR efficiently, as observed by GFP expression in approximately 50% of the cells, analysed by flow cytometry (Figure 2B). Intracelluar SAP protein staining also analysed by flow cytometry showed that G15 and G16 were able to restore SAP protein expression at the same efficiency indicated by GFP fluorescence, as anticipated from the co-translational design. However, HDR donors containing the separate GFP expression cassette (G7b/G14) were not able to restore SAP protein expression, potentially due to suppression of the *SH2D1A* promoter arising from the downstream EFS promoter (Villemure et al., 2001) (Figure 2B, Supplementary Figure S3).

To confirm this result, we performed further experiments in T cells from several healthy controls using the G7bc and G15 and G16 donors. Flow cytometry analysis confirmed high efficiency editing rates of 52, 50 and 18% respectively in CD3^+^ T cells (Figure 2C, Supplementary Figure S4A). T cell phenotype was further interrogated using flow cytometry markers CD4, CD8, CD45RA and CD62L. Importantly for an XLP therapeutic, the GFP + population contained both CD4^+^ and CD8^+^ T cells, and these populations were not skewed by the culture period or editing procedure (Figures 2D,E). Memory phenotype was similarly unaffected between edited and untouched controls (Figure 2F, Supplementary Figure S5).

To assess the degree to which HDR donors can replicate endogenous levels of SAP expression, we sorted GFP + cells from the bulk population and performed SAP staining. We found there was no significant difference in the level of SAP expression between untouched controls and cells that had been edited with either G15 or G16 HDR donor (Figure 2H). As expected, cells that had been edited with the G7bc donor and FACS-sorted had a complete knockout of SAP protein (Figure 2G, Supplementary Figure S6). From these data we concluded that the higher rates of editing achieved with the G15 donor, coupled with a highly favourable expression profile merited its use for all further experiments. To confirm that the SAP-containing donors were integrating at the correct loci we performed an in/out PCR, using a forward primer that binds upstream of the 5’ limit of the left homology arm and a reverse primer binding within the codon optimized SAP cDNA. As expected, a 953bp band was evident in cells treated with TALEN and G15 or G16 donors (Supplementary Figure S4B). We also performed ddPCR on the same amplicon, obtaining copy numbers (relative to an albumin control amplicon of similar length) consistent with the purity of cells selected using FACS-sorting (Supplementary Table S2).

### Optimisation of Transduction Conditions Allows Reduction of AAV6 HDR Template Dose

**FIGURE 3:**
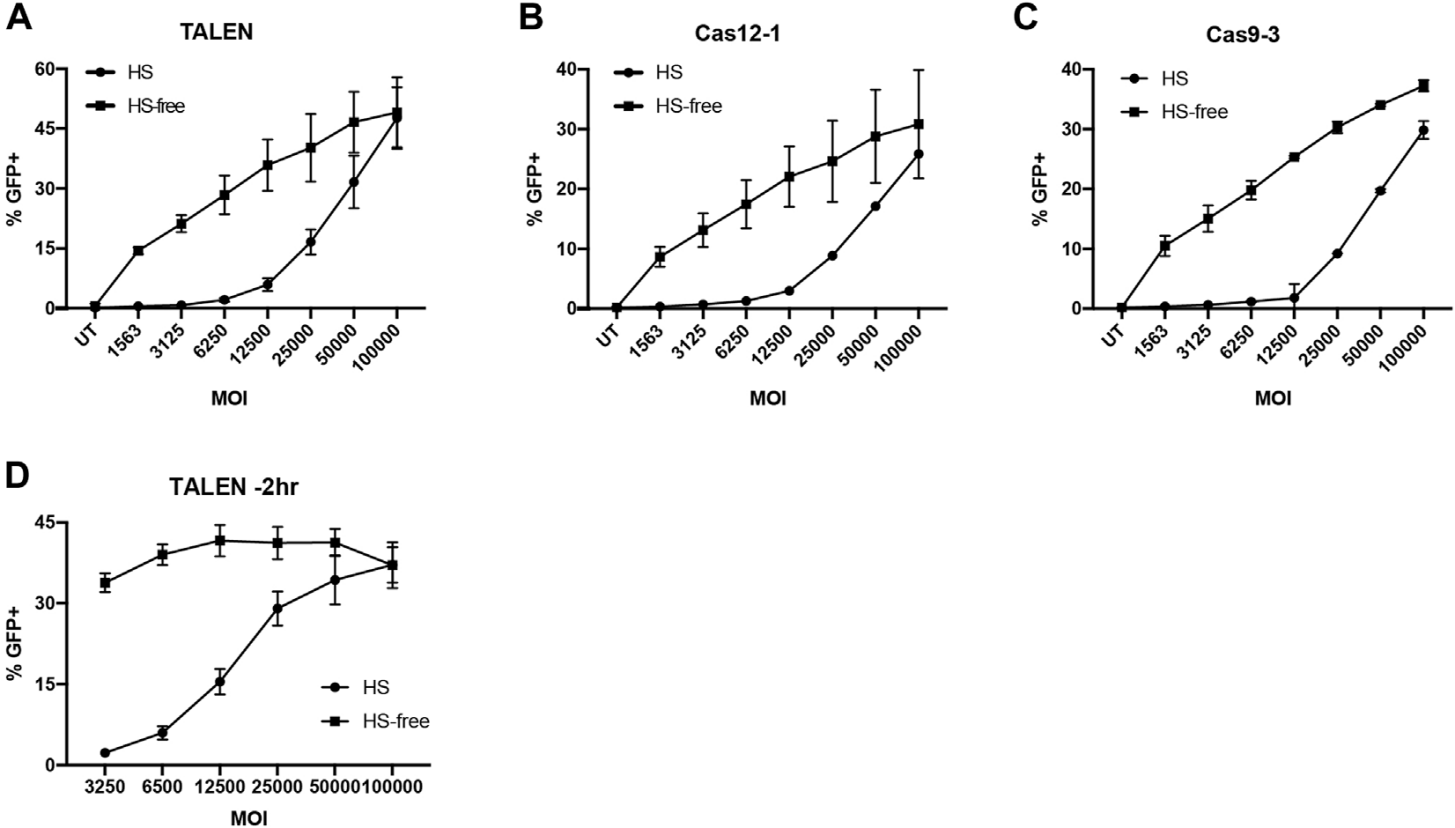
Serum free AAV6 transduction maintains HDR efficiency at reduced MOI. **(A–C)** %GFP + T cells transduced with G7bc AAV in HS or HS-free conditions 15 min post-nucleofection with **(A)** TALEN, **(B)** Cas12a-1 RNP or **(C)** Cas9-3 RNP (N = 2, mean, SD). **(D)** %GFP + T cells transduced with G7bc AAV at a range of MOIs 2h prior to TALEN nucleofection, in human serum (HS) or HS-free transduction conditions (n = 3, mean, SEM).

It has been reported in several studies that AAV6 is bound and neutralised by galectin 3 binding protein (G3BP) found in human and bovine serum, and that bovine serum-free culture conditions can enhance transduction of HSC and T cells (Denard et al., 2012; Song et al., 2013; Denard et al., 2018; Rogers et al., 2021). To test this in our hands, we transduced Jurkat cells at a range of doses (0–2 × 10^5^ GC/cell) in either culture media with or without foetal bovine serum (FBS). Those in FBS-free media were supplemented to full serum after 4 h. We observed significantly more GFP expression at reduced vector doses in the FBS-free conditions (Supplementary Figure S7). To investigate this in the context of a gene editing procedure we nucleofected T cells with TALEN, Cas9-3 or Cas12a-1 nucleases prior to transduction with AAV at a range of vector doses within 15 min, in T cell culture media with or without 5% human serum (HS). As before, those in low HS media were supplemented to full serum culture at 4 h. Across all nuclease platforms we found that cells transduced in the absence of HS had improved rates of HDR over a range of AAV6 vector doses (Figures 3A–C). Furthermore, we observed that the TALENs were able to mediate the highest rate of HDR up to 50%, while Cas9 and Cas12a had a similar efficiency with approximately 30% GFP positive cells.

When we tested HS-free transduction conditions 2 h prior to nucleofection with TALEN pairs, the effect was more pronounced, with no significant loss of GFP at 33-fold less AAV dose in the HS-free transduction conditions (Figure 3D, Supplementary Figure S8). As the TALEN pair offered preferential rates of HDR, particularly when using an HS-free transduction protocol, we used this platform for subsequent experiments in XLP patient T cells.

### SAP Protein Expression is Restored to the Level of Healthy Controls in Gene Edited XLP Patient T Cells

**FIGURE 4:**
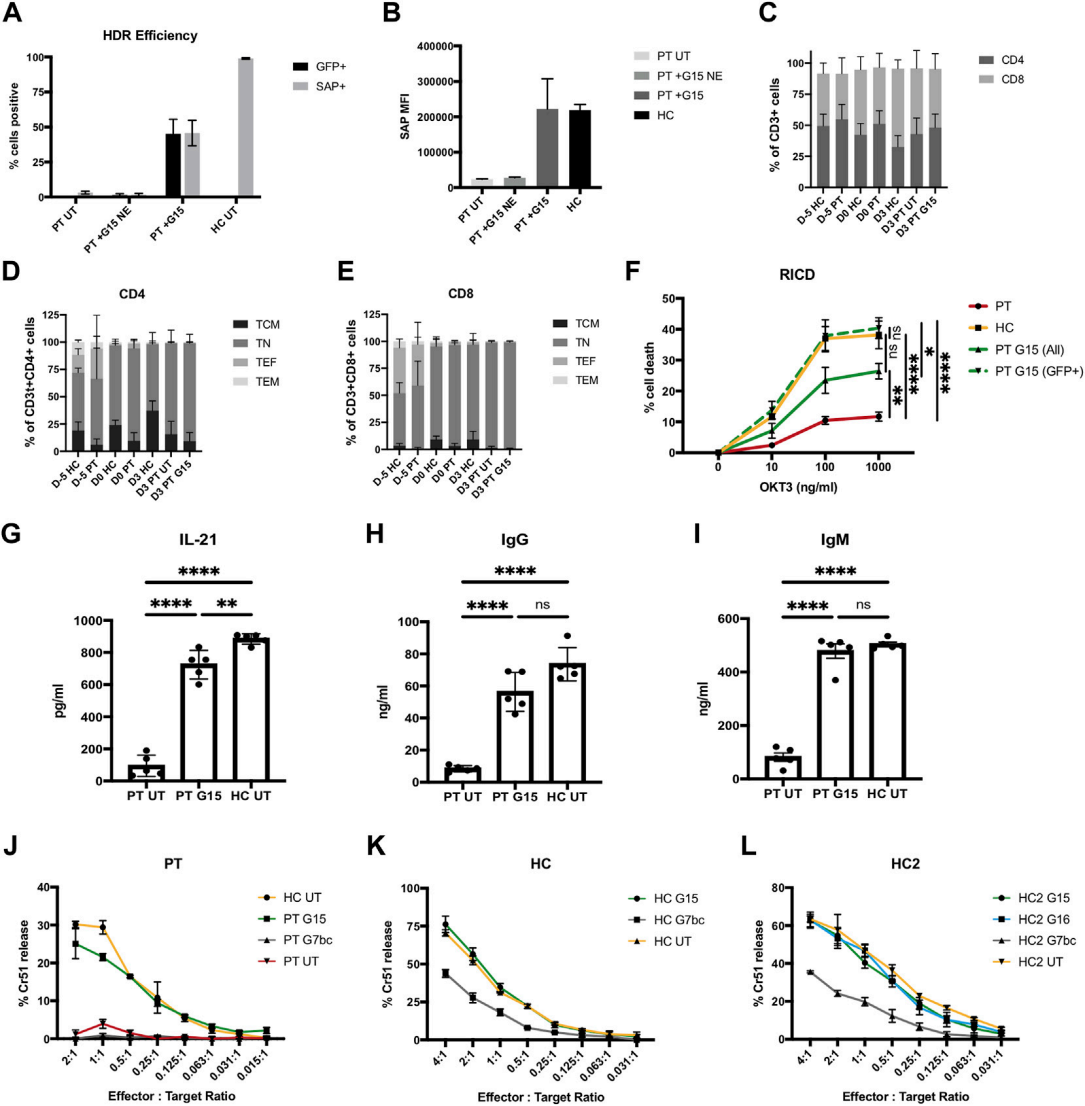
Genome editing restores SAP gene expression and function to XLP patient T cells. **(A–I)** PBMC harvested from 5 unrelated XLP patients stimulated and edited with TALEN and G15 HDR donor supplied 2 h prior to nucleofection in HS-free conditions, MOI2.5 × 10^4^. **(A)** %GFP+ (unstained cells, d3 post edit) and SAP+ (intracellularly stained, d6 post edit) lymphocytes analysed by flow cytometry; untouched patient (PT UT) and patient transduced without electroporation with nuclease (PT G15 NE), compared to cells receiving G15 and TALEN (PT + G15) and healthy control cells (HC). **(B)** MFI of SAP expressing lymphocytes. **(C)** % of CD3^+^ T cells expressing CD4 and CD8 on cell surface at point of thawing (D-5), editing (D0), and FACS analysis (D3) for healthy controls (HC), untreated patient (PT UT) or gene corrected with TALEN and G15 HDR donor (PT G15). **(D)** CD45RA CD62L T cell memory phenotype within the CD4^+^ T cell population. [E] CD45RA CD62L T cell memory phenotype within the CD8^+^ T cell population. [F] % cell death of lymphocytes restimulated with either 0, 10, 100, or 1,000 ng/ml OKT3 analysed by flow cytometry analysis by gating on the live bulk population (ALL) or live GFP + cells (GFP+), data normalised to non-restimulated condition with equation given in methods (mean, SEM). Stars denote Tukeys post-hoc test on one-way ANOVA performed on 1000 ng/ml OKT condition only: PT vs. HC, PT vs. PT G15 (GFP+), ****p < 0.0001; PT vs. PT G15 (All), **p = 0.0058; HC vs. PT G15 (All), ns *p* = 0.0660; HC vs. PT G15 (GFP+), ns *p* = 0.9524; PT G15 (All) vs. PT G15 (GFP+),*p = 0.0160. **(G–H)** Quantification of IL-21, IgG and IgM present in supernatant of 10 days co-culture of untreated patient cell (PT), the bulk population (unsorted) of gene edited patient cells (PT G15) or untreated healthy control (HC) naïve T cells with allogenic B cells in the presence of SEB, by means of ELISA (n = 5, mean, SEM). Stars denote Tukeys post-hoc test on one-way ANOVA **[G]** PT UT vs. PT G15/PT UT vs. HC UT, ****p < 0.0001; PT G15 vs. HC UT **p = 0.0081 [H] PT UT vs. PT G15/PT UT vs. HC UT, ****p < 0.0001; PT G15 vs. HC UT, ns *p* = 0.0727 **(I)** PT UT vs. PT G15/PT UT vs. HC UT, ****p < 0.0001; PT G15 vs. HC UT, ns *p* = 0.7534. **(J–L)** FACS-sorted (GFP+) CTL cytotoxic lymphocytes challenged against Chromium^51^-labelled EBV-B cell lymphoblastoid cell line targets at a series of effector:target cell ratios, graphs show % Chromium release compared to complete cell lysis, samples analysed in triplicate (n = 1, mean, SD). **(J)** Patient cells edited with G15 or G7bc HDR donor compared to untreated patient and healthy control. **(K)** Healthy control CTLs edited with either G15 or G7bc HDR donors and FACS-sorted on GFP + cells prior to assay, compared to untreated HC cells. **(L)** An additional healthy control CTL line (HC2) edited with G7bc, G15 and G16 HDR donors.

To ascertain if gene editing could restore SAP protein expression and SAP-dependent immune function to XLP patient T cells, PBMCs from 5 XLP patients were edited with TALEN and G15 HDR donor as before. HDR efficiency was measured by GFP expression, alongside T cell phenotyping markers on day 3 post edit. We achieved an average of 45 and 46% gene corrected lymphocytes, as indicated by GFP fluorescence and intracellular SAP staining respectively (Figure 4A, Supplementary Figure S9). Crucially, levels of SAP protein expression were highly similar to that of healthy controls. Furthermore, cells that had been transduced with HDR AAV but not electroporated had similar SAP protein to unedited patient cells indicating low levels of expression originating from non-integrated HDR vector (Figure 4B). Similarly to healthy T cell editing, we observed an equal distribution between CD4 and CD8 positive T cells (Figure 4C). Memory phenotyping markers within CD4 and CD8 T cell subsets were more varied in these samples, however, they remained broadly consistent between patient and healthy control groups throughout the experiment, and edited samples expanded to a similar extent as healthy and unedited patient cells (Figures 4D,E, Supplementary Figure S10). With this promising data we moved to test SAP-dependent immune function in three *in vitro* models.

### Gene Correction Restores SAP-dependent Immune Functions to XLP Patient T Cells

It has been shown that XLP T cells have reduced sensitivity to RICD, a process that in heathy individuals constrains immune responses by triggering apoptosis in cells restimulated through the TCR (Ruffo et al., 2016; Panchal et al., 2018b). To determine if gene edited cells can regain sensitivity to RICD, we cultured the gene edited T cells for 10 days, then restimulated with a range of concentrations of OKT3 antibody for 24 h before quantification of cell death using PI viability dye in flow cytometry following an established protocol (Katz and Snow, 2013). RICD in the bulk population was restored to approximately 50% that of healthy controls, matching the rate of HDR in the bulk population. When the analysis was performed only on corrected cells (GFP+), the amount of cell death equaled that of healthy controls, indicating sensitivity to RICD has been fully restored in the gene edited cells (Figure 4F, Supplementary Figure S11).

An absence of SAP in T_FH_ cells leads to humoral deficiencies, due to insufficient levels of T:B cell signaling in germinal centres. It has been shown that this interaction can be modelled *in vitro* by culturing naïve T cells with naïve B cells in the presence of SEB for 10 days before assessing levels of cytokine and immunoglobulin in the culture supernatant by ELISA (Ma et al., 2015; Panchal et al., 2018b). Naïve XLP patient T cells that had undergone a gene editing procedure showed restored T:B cell signaling, with B cell secretion of IgG and IgM corrected to the level of healthy controls, and significantly improved T_FH_ IL-21 secretion, compared to untreated patient cells (Figures 4G–I).

XLP patient T cells exhibit defective cytotoxicity against Epstein-Barr virus (EBV)-infected B cells (Sharifi et al., 2004; Dupré et al., 2005), which can contribute to the development of HLH. To test if gene editing could restore this function, we generated EBV-specific cytotoxic T lymphocytes (CTLs), by stimulating PBMC with irradiated LCL (Lymphoblastoid cell line, generated in house using EBV-B95.8 and healthy donor PBMC). Despite restimulated T cells showing significant loss of viability during the editing process, we were able to show in one patient sample that, even at low effector:target cell ratio, gene edited XLP patient T cells had similar killing activity to healthy control cells when challenged in a chromium^51^-release cytotoxicity assay (Figure 4J). To support this data, CTLs generated from healthy controls and edited with either G15 or G7bc HDR donors were GFP-sorted and similarly challenged. In two separate experiments, CTLs edited with SAP containing donor G15 showed equal killing to unedited healthy control CTLs, while those edited to create a SAP knockout using donor G7bc (Figure 2G) showed reduced killing activity (Figures 4J,L).

## Discussion

HSC therapy is widely used to treat primary immunodeficiencies, including XLP. However, GvHD remains a significant risk in the mismatched donor setting, leaving an unmet need that could be fulfilled by an autologous gene correction approach. We have previously shown that lentiviral vectors can be used to deliver a corrective copy of SAP cDNA into HSCs and T cells, to restore immune function *in vitro* and *in vivo* models of XLP. However, SAP has a tightly restricted profile that is challenging to replicate using this technology, which may be of particular importance when developing an HSC gene therapy approach. In this study, we aimed to use gene editing technology to create a site-specific insertion of SAP cDNA, hypothesising that this would harness more of the endogenous DNA regulatory mechanisms that govern SAP expression, to provide more physiological expression pattern and therefore a more optimal therapy in both the HSC and T cell setting.

Genome editing is centered on the creation of a site-specific DNA break, and there has been significant improvement in the specificity and efficiency of nucleases in recent years that has brought this technology into the clinic (Qasim et al., 2017; Frangoul et al., 2020). We opted to test three nuclease technologies, TALEN, CRISPR-S. p.HiFi Cas9 V3 and the more recently identified high activity mutant CRISPR-A.s.Cas12a Ultra (Zhang et al., 2021) to determine an optimal editor for the *SH2D1A* locus. We found all platforms capable of creating double strand DNA breaks at the *SH2D1A* locus at high efficiency. Cas9 creates a blunt cut, while TALENs and Cas12a create staggered cut that may more efficiently stimulate HDR (Zetsche et al., 2015). Despite generating INDELs at roughly half that of the Cas9 RNPs, our Cas12a RNPs were able to mediate similar levels of HDR as Cas9. In addition, TALENs created similar INDELs to Cas9 but were able to mediate more HDR, supporting this hypothesis.

We used targeted NGS to investigate on and off-target nuclease activity at sites predicted by *in silico* prediction software. On target amplicons confirmed the highly efficiency genome modification mediated by all three nuclease platforms. Modifications at off-target loci occurred at low frequency, and were not detected in exons, indeed the nearest exon boundary was over 450bp away for Cas9-3 OT1 (*RPTOR*) and over 20 kb for TALEN OT2 (*TET1*), suggesting the risk of mutagenesis in coding regions remains low. However, it may be possible to mitigate the TALEN off target activity by using a recently described *FokI* mutant (Miller et al., 2019). Miller and others identified several single residue substitutions in *Fok*I that exhibited a significant reduction in off-target activity, up to 1000-fold in some case. The substitutions lead to a reduction in catalytic rate, allowing dissociation from mismatched targets before cleavage occurs. Although several variants may need to be tested for each locus, this technology could be readily transferred to the TALEN platform (Miller et al., 2019).

It has previously been shown that using a RNP delivery format and a high-fidelity Cas9 variant can reduce off target cutting activity of Cas9, yet, despite adopting these technologies, we found evidence of low level off-target cutting at the Cas9-3 OT1 site. Mitigation may be possible through introducing internal 2′-O-methyl-3′-phosphonoacetate modifications to gRNA, however, in the absence of established design principles, optimal modifications must be determined empirically for each guide (Ryan et al., 2018; Vakulskas and Behlke, 2019) Further Cas9 protein engineering is likely to identify additional variants with improved target specificity. Indeed, a recent study used kinetics-guided cryo-electron microscopy to identify a kinked duplex structure, formed between DNA and gRNA with mismatches only at the PAM-distal positions 18–20, that supports an active Cas9 configuration (Bravo et al., 2022). A targeted mutation (creating SuperFi-Cas9) was able ablate this structure and reduce off target activity of sgRNA with PAM-distal mismatcheds. Although at an early stage, these studies could be particularly useful to our application, as Cas9-3 OT1 mismatches occur at gRNA positions 19 and 20.

As both *TET1* and *RPTOR* are important for haematopoiesis and T-cell differentiation and function, there is a possibility of mutation of intronic regulatory signals, further studies of mutations at these OT loci may be required, alongside unbiased genome-wide screening for additional loci not predicted in silico, prior to translation to the clinic (Ko et al., 2015; Wang et al., 2016), Little is known about mechanisms involved in SAP gene expression. Consensus binding site for Ets-1 and Ets-2 have been identified as being important for core promoter activity (Okamoto et al., 2004), and we have previously attempted to generate lentiviral vectors incorporating this motif by cloning proximal sections of the promoter. The expression output of these vectors was significantly below physiological levels, indicating that other motifs must be important (Panchal et al., 2018b). Our exon1 gene editing approach allows for the all the upstream promoter regulatory mechanisms to be preserved. At the 3′end of SH2D1A encoding mRNA, RNA binding protein sites have been identified that could have a role in regulating SH2D1A RNA stability in the cells (Okamoto et al., 2004). In addition, several studies have validated microRNA binding sites in the SAP3′UTR that have a role in regulation of SAP (Ding et al., 2012), most prominently miRNA-31 (Heide et al., 2016; Ripamonti et al., 2017), which is known to play a role in regulating IL-2 secretion (Fan et al., 2012) and IFN signalling in CD8 T cells (Moffett et al., 2017). To take advantage of these regulatory motifs, we generated an HDR donor that incorporated the full length 3′UTR, as this strategy has been shown to improve the expression profile in other T cell editing studies (Hubbard et al., 2016). However, we did not see any notable improvement in our studies but were hampered by reduced HDR efficiency. AAV vector titre was also reduced, likely due to the full-length UTR slightly exceeding the packaging capacity of AAV particles, leading to packaging of incomplete genomes. Further studies using shorter sections of UTR, looking in different T cell populations at different time points post-activation could provide more insight into the importance of these motifs. Our construct using the woodchuck post transcriptional element (WPRE) gave rise to expression equal to that of healthy controls, and furthermore, is widely used in gene therapy vectors for primary immune deficiency (Gaspar et al., 2006; Marangoni et al., 2009; Kohn et al., 2020), presenting a clearer path to clinical translation.

We were motivated to optimise the transduction protocols by the high cost associated with performing gene editing at clinical scale. It has been reported that AAV6 is bound and precipitated by G3BP in FBS and HS (Denard et al., 2012) leading to a reduction in transduction efficiency. Here, we show that transducing T cells in media without HS leads to significantly improved gene editing at reduced viral doses, particularly when transduction is performed prior to nucleofection. Similar findings have recently been reported where T cells are cultured using FBS, supporting this approach (Wang et al., 2015; Rogers et al., 2021). In our study, the serum-free culture period had no effect on T cell phenotype markers. This approach could offer significant savings in viral production costs needed for each clinical product. However, further optimisations may be possible, either at the level of AAV transduction—such as human serum albumin (HSA) or polyvinyl alcohol (PVA) that have increased HSC transduction (Wang et al., 2017; Yang et al., 2020), or at the level of the proteins involved in DNA repair pathway by using small molecules (Li et al., 2017; Ma et al., 2018; Bischoff et al., 2020).

In the absence of SAP, XLP patient T cells exhibit deficits in several immune functions, including reduced sensitivity to RICD, insufficient T:B cell signalling and a lack a cytotoxicity against EBV-infected B cell targets. Here, we have shown for the first time that gene editing XLP patient T cells can restore SAP-dependent immune functions to the level of healthy control cells in assays that model these deficits. Although ultimately the number of corrected T cells needed for clinical benefit can only be fully determined in clinical trial, we are guided by patients that show somatic reversion of mutations. Such patients may have a SAP replete population as low as 5–8%, which leads to significantly prolonged survival without transplant (up to 40 years reported) suggesting a significant reduction in the risk of developing HLH and possibly malignancy, even with low levels of corrected cells (Palendira et al., 2012; Hoshino et al., 2018). We have also seen in our previous work using a lentiviral strategy for T cell correction in an XLP murine modal that 20–40% correction can lead to complete functional recovery (Panchal et al., 2018b). Drawing on these results, the rates of HDR reached in this work (30–50%) would be at a level that could restore a clinically relevant level of immune function in patients.

However, while a T cell product could treat several of the most severe clinical manifestations of XLP and offer a life-saving bridge to HSCT, studies have shown that NKT cells (which are absent in XLP patients) have an important role in tumour surveillance (Das et al., 2013; Weng et al., 2014), and SAP-replete NK cells can aid with clearing EBV-infected cells (Parolini et al., 2000). Here we have demonstrated across different gene editing platforms that it is possible to target the SAP gene locus with high levels of efficiency and specificity and promote corrective HDR in the context of primary T cells, leading to functional rescue. We are now transferring these technologies to HSCs with the aim of developing a gene edited HSC therapy for patients with XLP lacking a suitable donor for HSCT.

## Materials and Methods

### Human Samples

Informed consent was obtained from all human subjects, including 6 unrelated XLP patients that have confirmed mutations in SH2D1A. Pt1—exon 2 deletion; Pt2—exon 2 deletion; Pt3—c.163c > t; Pt4—c.163C > T, p.Arg55X; Pt5 –exon 2-4 deletion; Pt6—exon1 deletion, which was not amenable to gene editing. Pt1 was used for testing HDR donor constructs and EBV-B cell cytotoxicity, Pts1-5 we were used in T:B co-culture assay and analysis of MFI in corrected cells, Pt1,2,3,5 corrected cells were used for RICD assay, Pt1-6 as untreated (UT) controls.

### Cell Culture

Jurkat T cells and lymphoblastoid cell lines were cultured in RPMI containing 10% Foetal bovine serum (FBS) and 1% Penicillin-streptomycin (pen-strep) and passaged twice weekly using 1:10 dilution. HEK293Ts were maintained in DMEM supplemented with 10% FBS and 1% pen-strep and passaged twice weekly. Cells were washed with PBS and released from the culture flask with Trypsin-EDTA (all reagents ThermoFisher Scientific) before collection and neutralisation in complete DMEM and seeding back into culture flask at a 1:10 dilution.

### T Cell Stimulation and Culture

Human peripheral blood mononuclear cells (PBMC) were harvested from whole blood using Ficoll-Paque density centrifugation (GE Healthcare). PBMC were cultured in TexMACS™ Medium (Miltneyi) supplemented with 5% human serum (Sigma) and 1% pen-strep. PBMC were stimulated with Human T-Activator CD3/CD28 Dynabeads (Gibco) at 1:1 ratio, in the presence of 100 U/ml IL-2 (Proleukin) in G-Rex^®^ 24 plates (Wilson Wolf).

### TALEN mRNA Synthesis

TALEN pairs were identified and constructed as previously described (Mussolino et al., 2011). Plasmid constructs were linearised at the 3′ of the expression cassette using restriction enzyme digest, then purified (Qiagen). mRNA was produced using the T7 mScript™ Standard mRNA Production System according to manufacturer’s protocol. Briefly, linearised DNA template was transcribed, treated with DNAse1 and purified (RNeasy Mini-kit, Qiagen), before further reactions for addition of a polyA tail using the supplied enzymes, and Cap 1 structure capping. After a further purification, mRNA integrity was assessed using TapeStation (Agilent) and quantified on NanoDrop Microvolume Spectrophotometer. Left and right TALEN arms were combined at 1:1 ratio, aliquoted (7.5 µg each arm) and stored at −20°C.

### CRISPR-Cas9 and CRISPR-Cas12a Design and RNP Assembly

Potential Cas9 and Cas12a target sites were identified using Benchling online software (www.benchling.com). Cas9 (Alt-R^®^ S. p. HiFi Cas9 Nuclease V3, IDT) and Cas12a (Alt-R^®^ A.s. Cas12a (Cpf1) Ultra, IDT) proteins were complexed to their respective RNA guides (Cas9 synthetic gRNA, Merck; Cas12a crRNA, IDT) at a protein:guide molar ratio of 1:2.5 (Vakulskas et al., 2018) and 1:2 respectively, at room temperature for 10 min immediately prior to nucleofection.

### T Cell Editing

T cells were cultured as described above. Dynabeads were removed using a DynaMag™-15 Magnet (Invitrogen), and cells washed in PBS and counted. For serum-free transduction prior to nucleofection, cells were washed again and resuspended in TexMACs media with pen-strep and IL-2 but without human serum. Nucleases were delivered into cells *via* electroporation using the Lonza 4D nucleofector, buffer P3, program EO-115 - typically 1.5-3 million cells in the 1 ml cuvette, or 0.5-1 million the 20 µl cuvette.

### Determining Nuclease Efficiency Using TIDE/ICE Analysis

Genomic DNA was harvested from edited cells at day 5 post nucleofection (Qiagen). PCR amplicons were generated using the following primer pair for all nucleases (Fwd: TGG​CCT​CTG​AGT​AAA​CCG​CA, Rev: AGC​GAG​GGA​TTG​AGG​CGA​AA, product length: 718bp, Tm: 69°C) using Q5 polymerase (NEB). After PCR purification (Qiagen), amplicons were sanger sequenced (Eurofins genomics) using the forward primer. The resulting ab.1 file was the input to the online TIDE tool (Brinkman et al., 2014) (TALENs), or ICE software (Cas9/Cas12a, Synthego) which generated the % modification score.

### Assessment of Cutting Activity at Predicted off-Target Loci

The top 10 most highly predicted loci for off-target nuclease activity of Cas9-3 and Cas12a-1 were identified by Benchling online software, while TALEN sites were predicted using PROGNOS (http://bao.rice.edu/cgi-bin/prognos/prognos.cgi) (Fine et al., 2014). PCR amplicons were designed to generate 150-200bp with the expected cut site in the centre. DNA from male healthy donor T cells edited with each nuclease platform (alongside untreated controls) was extracted at day 3 post nucleofection (Qiagen) and used as a template for on-target and off-target PCR reactions. The amplicons were then prepared for Illumina next generation sequencing by performing end repair, adapter ligation and bar coding using the NEBNext^®^ UltraTM II DNA Library Prep Kit (NEB) according to the manufacturer’s instructions. Libraries were quantified using the ddPCR™ Library Quantification Kit for Illumina TruSeq (Biorad), before sequencing using MiSeq Reagent Kit v2, 500cycles on an Illumina MiSeq platform (Illumina). The generated paired-end reads were processed using the command line version of the CRISPResso2 pipeline (Clement et al., 2019), obtained editing frequencies were compared to the untreated control samples using a one-sided Fisher’s exact test. *, **, and *** indicate *p* < 0.05, *p* < 0.01, *p* < 0.001, respectively.

### AAV6 Production and Purification

An AAV2 genome plasmid was kindly provided by Professor Amit Nathwani (UCL), and the pDGM6 (RRID:Addgene_110660) AAV6 packaging plasmid by the Russel lab (University of Washington). Homology arms were amplified from healthy control genomic DNA, both right and left 850bp long in all donor constructs (Right homology arm ChrX:124345793-124346642, left homology arm ChrX:124346644-124347493). Constructs were cloned using HiFi assembly (NEB), allowing the left homology arm sequence to run directly into a codon optimised SAP cDNA without restriction enzyme sequences. SH2D1A 3′UTR sequences were also amplified from genomic DNA, all other elements were amplified from previously described lentiviral plasmids (Rivat et al., 2013; Panchal et al., 2018b).

AAV6 particles were produced in HEK293T cells *via* co-transfection of pDGM6 and the HDR genome plasmid using polyethylenimine (PEI, Sigma-Aldrich) 24 h after plating in complete DMEM media (DMEM (Gibco) 10%FBS, 1% penstrep). Media was replaced after 4 h and replaced again after 24 h with 2% DMEM. After a further 48 h, the cells were released into the media by scraping, before centrifuging to separate cell pellet and culture supernatant for processing separately. AAV6 particles were precipitated from cell media using ammonium sulphate on ice for 30min before collection by centrifugation and resuspended in TD buffer (1xPBS, 1mM MgCl_2_, 2.5 mM KCl). The cell pellet was resuspended in TD and freeze/thaw ×4 in the presence of 0.5% deoxycholic acid (VWR), before centrifugation and harvesting the supernatant. Both fractions were incubated with benzonase 50 U/ml (Novagen) before combining prior to iodixanol-gradient centrifugation. AAV6 particles were harvested from the 40–60% gradient interface and stored at 4°C. Titration was performed using the QuickTiter™ AAV Quantitation Kit (Cell Biolabs).

### In/out PCR and ddPCR

Primers for detection of G15 and G16 donors at the SH2D1A locus were designed using the NIH Primer-BLAST tool (Ye et al., 2012) (Fwd: TGG​ACA​AAA​TGC​TGA​AAG​GTG​G; Rev: GTC​TCT​CTG​CTG​ATC​TTG​CCG, amplicon length 953bp, Tm: 64°C). For ddPCR, the same primers were used with the addition of a probe CCA​GGG​CTC​CGG​AGT​CAG​GC (5′6-FAM, Internal ZEN and 3′ Iowa Black FQ, IDT). The genomic reference amplicon primers targeted albumin (Fwd GCT​GTC​ATC​TCT​TGT​GGG​CTG, Rev CAC​AAA​TTT​GGA​AAC​AGA​ACA​GGC​ATT, amplicon length 1035bp) and probe CCT​GTC​ATG​CCC​ACA​CAA​ATC​TCT​CC (5′HEX, Internal ZEN and 3′ Iowa Black FQ, IDT). Droplets were generated and analysed according to the manufacturer’s instructions (QX200 system, Bio-Rad). The cycling conditions were (95°C 10 min initiation, 50x (94°C 1min, 60°C 30 s, 72°C 6 min) 98°C 10 min, store 12 C).

### Detection of SAP Protein Expression by Intracellular Staining and Flow Cytometry

Intracellular staining of SAP was performed using the IntraPrep Permeabilizaton Reagent (Beckman). The primary antibody was either mouse anti-human SH2D1A antibody (Clone 1C9 - Abnova Cat# H00004068-M01, RRID:AB_425532), or isotype control (Novus Cat# NB110-7082, RRID:AB_790752). The secondary was Goat anti-mouse polyclonal immunoglobulins RPE Goat F (ab’)2 (Dako).

### T Cell Phenotyping

The following T cell phenotyping panel was used to determine Tcm, Tem, Tscm and Tnaive populations - CD3 PE-Cy7 (Clone UCHT1), CD4 APC-Cy7 (Clone RPA-T4), CD8 APC (Clone RPA-T8), CD62L BV510 (Clone DREG-56), CD45RA BV650 (Clone HI100), CD95 BV711 (Clone DX2) (BD Biosciences).

### T:B Cell Co-Culture Assay

PBMC were stimulated and edited as described and cultured for 7 days, before naïve T cell magnetic bead selection according to the manufacturers protocol (Miltneyi). T cells were plated at 5.0 × 10^4^ cells/well in a round-bottom 96 well plate. Naïve B cells were isolated from tonsillar mononuclear cells (Milteny) and added to the T cells at a 1:1 ratio, in addition to 150 ng/ml staphylococcal enterotoxin B (SEB). Cells were co-cultured for 10 days, before the harvesting the supernatant for ELISA analysis of IL-21, IgG and IgM (eBioscience).

### RICD Assay

RICD was assessed using an established protocol (Katz and Snow, 2013). Briefly, edited T cells were cultured for 10 days, before washing with PBS, counting and plating at 5.0 × 10^4^ cells/well in triplicate in a 96 well plate in 100 µl media. Dilutions of OKT3 antibody (Tonbo Biosciences) were prepared at 2000 ng/ml, 200 ng/ml, and 20 ng/ml, and 100 µl added to the cells to make final concentrations of 1000 ng/ml, 100 ng/ml and 10 ng/ml in the wells.

After 24 h, PI was added (final concentration 1 μg/ml) before running a fixed volume of cell suspension from each well in flow cytometry (Cytoflex, Beckman). The data was analysed by determining the number of live cells (PI-) and comparing unstimulated controls to stimulated conditions by using the formula: % cell loss = [1-(# PI- restimulated cells/# PI- untreated cells)]x100.

### Generation of EBV-B Cell Specific Cytotoxic T Lymphocytes (CTLs) and Chromium^51^-Release Cytotoxicity Assay

Lymphoblastoid cell lines (LCL) were previously created from EBV-seropositive healthy donors using EBV B95.8 supernatant (Ricciardelli et al., 2014; Panchal et al., 2018b). EBV-CTLs were generated by stimulating PBMC with 40Gy-irradiated LCL, initially at 40:1 PBMC:LCL ratio in the absence of IL-2 for 10 days, then subsequently at weekly intervals at 4:1 ratio in the presence of 100 U/ml IL-2. CTLs were edited after the third stimulation and sorted by flow cytometry for corrected cells (GFP+) before a fourth restimulation. To test cytotoxic capability, LCLs were labelled with Chromium^51^ (Na_2_^51^CrO_4_, Perkin Elmer) for 1 h at 37°C. An initial effector:target ratio was determined based on available effector cell numbers, then a twofold serial dilution was used to determine cytotoxic range. Labelled target cells were washed and added to the plate (5000/well) for 4 h at 37°C. 50 µl culture supernatant was taken and added to 150 µl scintillation fluid and incubated for 12 h at room temperature. Cr^51^ release was determined using a beta counter (PerkinElmer).

### Statistical Analysis

Statistical analysis was performed with Graphpad Prism 9 software.

Data is presented as the mean ± SEM or SD as denoted in the figure legend. Statistical analysis was performed using Prism 9 software (Graphpad Software Inc.), details of statistical tests used, including all *p* values are indicated in the relevant figure legend.

## Data Availability

The original contributions presented in the study are publicly available. This data can be found here: https://www.ncbi.nlm.nih.gov/sra/PRJNA799193.

## References

[B1] Bischoff N. Wimberger S. Maresca M. Brakebusch C. (2020). Improving Precise CRISPR Genome Editing by Small Molecules: Is There a Magic Potion? Cells 9, 1318. 10.3390/cells9051318 PMC7291049 32466303

[B2] Booth C. Gilmour K. C. Veys P. Gennery A. R. Slatter M. A. Chapel H. (2011). X-Linked Lymphoproliferative Disease Due to SAP/SH2D1A Deficiency: A Multicenter Study on the Manifestations, Management and Outcome of the Disease. Blood 117, 53–62. 10.1182/blood-2010-06-284935 20926771 PMC3374620

[B3] Bravo J. P. K. Liu M.-S. Hibshman G. N. Dangerfield T. L. Jung K. McCool R. S. (2022). Structural Basis for Mismatch Surveillance by CRISPR-Cas9. Nature 603, 343–347. 10.1038/s41586-022-04470-1 35236982 PMC8907077

[B4] Brinkman E. K. Chen T. Amendola M. van Steensel B. (2014). Easy Quantitative Assessment of Genome Editing by Sequence Trace Decomposition. Nucleic Acids Res. 42, e168. 10.1093/nar/gku936 25300484 PMC4267669

[B5] Cannons J. L. Tangye S. G. Schwartzberg P. L. (2011). SLAM Family Receptors and SAP Adaptors in Immunity. Annu. Rev. Immunol. 29, 665–705. 10.1146/annurev-immunol-030409-101302 21219180

[B6] Clement K. Rees H. Canver M. C. Gehrke J. M. Farouni R. Hsu J. Y. (2019). CRISPResso2 Provides Accurate and Rapid Genome Editing Sequence Analysis. Nat. Biotechnol. 37, 224–226. 10.1038/s41587-019-0032-3 30809026 PMC6533916

[B7] Comte D. Karampetsou M. P. Humbel M. Tsokos G. C. (2018). Signaling Lymphocyte Activation Molecule Family in Systemic Lupus Erythematosus. Clin. Immunol. 204, 57–63. 10.1016/j.clim.2018.11.001 30415085

[B73] Das R. Bassiri H. Guan P. Wiener S. Banerjee P. P. Zhong M. C. (2013). The Adaptor Molecule SAP Plays Essential Roles During Invariant NKT Cell Cytotoxicity and Lytic Synapse Formation. Blood 121 (17), 3386–95. 10.1182/blood-2012-11-468868 23430111 PMC3637014

[B8] Denard J. Beley C. Kotin R. Lai-Kuen R. Blot S. Leh H. (2012). Human Galectin 3 Binding Protein Interacts with Recombinant Adeno-Associated Virus Type 6. J. Virol. 86, 6620–6631. 10.1128/jvi.00297-12 22496229 PMC3393578

[B9] Denard J. Rouillon J. Leger T. Garcia C. Lambert M. P. Griffith G. (2018). AAV-8 and AAV-9 Vectors Cooperate with Serum Proteins Differently Than AAV-1 and AAV-6. Mol. Ther. - Methods Clin. Dev. 10, 291–302. 10.1016/j.omtm.2018.08.001 30155509 PMC6111067

[B10] Ding S. Liang Y. Zhao M. Liang G. Long H. Zhao S. (2012). Decreased microRNA-142-3p/5p Expression Causes CD4+ T Cell Activation and B Cell Hyperstimulation in Systemic Lupus Erythematosus. Arthritis Rheum. 64, 2953–2963. 10.1002/art.34505 22549634

[B11] Dragovich M. A. Mor A. (2018). The SLAM Family Receptors: Potential Therapeutic Targets for Inflammatory and Autoimmune Diseases. Autoimmun. Rev. 17, 674–682. 10.1016/j.autrev.2018.01.018 29729453 PMC6508580

[B12] Duprè L. Andolfi G. Tangye S. G. Clementi R. Locatelli F. Aricò M. (2005). SAP Controls the Cytolytic Activity of CD8+ T Cells against EBV-Infected Cells. Blood 105, 4383–4389. 10.1182/blood-2004-08-3269 15677558

[B13] Fan W. Liang D. Tang Y. Qu B. Cui H. Luo X. (2012). Identification of microRNA-31 as a Novel Regulator Contributing to Impaired Interleukin-2 Production in T Cells from Patients with Systemic Lupus Erythematosus. Arthritis Rheum. 64, 3715–3725. 10.1002/art.34596 22736314

[B14] Fine E. J. Cradick T. J. Zhao C. L. Lin Y. Bao G. (2014). An Online Bioinformatics Tool Predicts Zinc finger and TALE Nuclease Off-Target Cleavage. Nucleic Acids Res. 42, e42. 10.1093/nar/gkt1326 24381193 PMC3973315

[B15] Frangoul H. Altshuler D. Cappellini M. D. Chen Y.-S. Domm J. Eustace B. K. (2020). CRISPR-Cas9 Gene Editing for Sickle Cell Disease and β-Thalassemia. N. Engl. J. Med. 384, 252–260. 10.1056/nejmoa2031054 33283989

[B16] Gartshteyn Y. Askanase A. D. Mor A. (2021). SLAM Associated Protein Signaling in T Cells: Tilting the Balance toward Autoimmunity. Front. Immunol. 12, 654839. 10.3389/fimmu.2021.654839 33936082 PMC8086963

[B17] Gaspar H. B. Bjorkegren E. Parsley K. Gilmour K. C. King D. Sinclair J. (2006). Successful Reconstitution of Immunity in ADA-SCID by Stem Cell Gene Therapy Following Cessation of PEG-ADA and Use of Mild Preconditioning. Mol. Ther. 14, 505–513. 10.1016/j.ymthe.2006.06.007 16905365

[B18] Geng L. Yang J. Tang X. Peng H. Tian J. Hu Z. (2021). SLAM/SAP Decreased Follicular Regulatory T Cells in Patients with Graves' Disease. J. Immunol. Res. 2021, 1–11. 10.1155/2021/5548463 PMC8079219 33987447

[B19] Genovese P. Schiroli G. Escobar G. Di Tomaso T. Firrito C. Calabria A. (2014). Targeted Genome Editing in Human Repopulating Haematopoietic Stem Cells. Nature 510, 235–240. 10.1038/nature13420 24870228 PMC4082311

[B20] Gillmore J. D. Gane E. Taubel J. Kao J. Fontana M. Maitland M. L. (2021). CRISPR-Cas9 *In Vivo* Gene Editing for Transthyretin Amyloidosis. N. Engl. J. Med. 385, 493–502. 10.1056/nejmoa2107454 34215024

[B21] Hale J. S. Youngblood B. Latner D. R. Mohammed A. U. R. Ye L. Akondy R. S. (2013). Distinct Memory CD4+ T Cells with Commitment to T Follicular Helper- and T Helper 1-Cell Lineages Are Generated after Acute Viral Infection. Immunity 38, 805–817. 10.1016/j.immuni.2013.02.020 23583644 PMC3741679

[B22] Heide V. V. D. Möhnle P. Rink J. Briegel J. Kreth S. (2016). Down-regulation of MicroRNA-31 in CD4+ T Cells Contributes to Immunosuppression in Human Sepsis by Promoting TH2 Skewing. Anesthesiology 124, 908–922. 10.1097/aln.0000000000001031 26978146

[B23] Hoshino A. Yang X. Tanita K. Yoshida K. Ono T. Nishida N. (2018). Modification of Cellular and Humoral Immunity by Somatically Reverted T Cells in X-Linked Lymphoproliferative Syndrome Type 1. J. Allergy Clin. Immun. 143, 421–424. 10.1016/j.jaci.2018.07.044 30342818

[B24] Hubbard N. Hagin D. Sommer K. Song Y. Khan I. Clough C. (2016). Targeted Gene Editing Restores Regulated CD40L Function in X-Linked Hyper-IgM Syndrome. Blood 127, 2513–2522. 10.1182/blood-2015-11-683235 26903548

[B25] Katz G. Snow A. L. (2013). Immune Homeostasis, Methods and Protocols. Methods Mol. Biol. 979, 15–25. 10.1007/978-1-62703-290-2 23397384

[B26] Ko M. An J. Rao A. (2015). DNA Methylation and Hydroxymethylation in Hematologic Differentiation and Transformation. Curr. Opin. Cel Biol. 37, 91–101. 10.1016/j.ceb.2015.10.009 PMC4688184 26595486

[B27] Kohn D. B. Booth C. Booth C. Kang E. M. Pai S.-Y. Shaw K. L. (2020). Lentiviral Gene Therapy for X-Linked Chronic Granulomatous Disease. Nat. Med. 26, 200–206. 10.1038/s41591-019-0735-5 31988463 PMC7115833

[B28] Kuo C. Y. Long J. D. Campo-Fernandez B. de Oliveira S. Cooper A. R. Romero Z. (2018). Site-Specific Gene Editing of Human Hematopoietic Stem Cells for X-Linked Hyper-IgM Syndrome. Cel Rep. 23, 2606–2616. 10.1016/j.celrep.2018.04.103 PMC6181643 29847792

[B29] Li G. Zhang X. Zhong C. Mo J. Quan R. Yang J. (2017). Small Molecules Enhance CRISPR/Cas9-mediated Homology-Directed Genome Editing in Primary Cells. Sci. Rep. 7, 8943. 10.1038/s41598-017-09306-x 28827551 PMC5566437

[B30] Liu B. Zeng L. Shao Y. Fu R. (2021). Expression and Function of SLAMF6 in CD8+ T Lymphocytes of Patients with Severe Aplastic Anemia. Cell Immunol. 364, 104343. 10.1016/j.cellimm.2021.104343 33774556

[B31] Ma C. S. Pittaluga S. Avery D. T. Hare N. J. Maric I. Klion A. D. (2006). Selective Generation of Functional Somatically Mutated IgM+CD27+, but Not Ig Isotype-Switched, Memory B Cells in X-Linked Lymphoproliferative Disease. J. Clin. Invest. 116, 322–333. 10.1172/jci25720 16424938 PMC1332028

[B69] Ma C. S. Wong N. Rao G. Avery D. T. Torpy J. Hambridge T. (2015). Monogenic Mutations Differentially Affect the Quantity and Quality of T Follicular Helper Cells in Patients With Human Primary Immunodeficiencies. J. Allergy Clin. Immunol. 136 (4), 993–1006.e1. 10.1016/j.jaci.2015.05.036 26162572 PMC5042203

[B32] Ma X. Chen X. Jin Y. Ge W. Wang W. Kong L. (2018). Small Molecules Promote CRISPR-Cpf1-Mediated Genome Editing in Human Pluripotent Stem Cells. Nat. Commun. 9, 1303. 10.1038/s41467-018-03760-5 29610531 PMC5880812

[B33] Malaer J. D. Marrufo A. M. Mathew P. A. (2019). 2B4 (CD244, SLAMF4) and CS1 (CD319, SLAMF7) in Systemic Lupus Erythematosus and Cancer. Clin. Immunol. 204, 50–56. 10.1016/j.clim.2018.10.009 30347240

[B34] Marangoni F. Bosticardo M. Charrier S. Draghici E. Locci M. Scaramuzza S. (2009). Evidence for Long-Term Efficacy and Safety of Gene Therapy for Wiskott-Aldrich Syndrome in Preclinical Models. Mol. Ther. 17, 1073–1082. 10.1038/mt.2009.31 19259069 PMC2835187

[B35] Mehrle S. Frank S. Schmidt J. Schmidt-Wolf I. G. Märten A. (2005). SAP and SLAM Expression in Anti-CD3 Activated Lymphocytes Correlates with Cytotoxic Activity. Immunol. Cel Biol 83, 33–39. 10.1111/j.1440-1711.2005.01302.x10.1111/j.1440-1711.2004.01302.x 15661039

[B36] Miller J. C. Patil D. P. Xia D. F. Paine C. B. Fauser F. Richards H. W. (2019). Enhancing Gene Editing Specificity by Attenuating DNA Cleavage Kinetics. Nat. Biotechnol. 37, 945–952. 10.1038/s41587-019-0186-z 31359006

[B37] Moffett H. F. Cartwright A. N. R. Kim H.-J. Godec J. Pyrdol J. Äijö T. (2017). The microRNA miR-31 Inhibits CD8+ T Cell Function in Chronic Viral Infection. Nat. Immunol. 18, 791–799. 10.1038/ni.3755 28530712 PMC5753758

[B38] Mussolino C. Morbitzer R. Lütge F. Dannemann N. Lahaye T. Cathomen T. (2011). A Novel TALE Nuclease Scaffold Enables High Genome Editing Activity in Combination with Low Toxicity. Nucleic Acids Res. 39, 9283–9293. 10.1093/nar/gkr597 21813459 PMC3241638

[B39] Okamoto S. Ji H. Howie D. Clarke K. Gullo C. Manning S. (2004). Expression of theSH2D1A Gene Is Regulated by a Combination of Transcriptional and Post-Transcriptional Mechanisms. Eur. J. Immunol. 34, 3176–3186. 10.1002/eji.200324755 15459902

[B40] Palendira U. Low C. Bell A. I. Ma C. S. Abbott R. J. M. Phan T. G. (2012). Expansion of Somatically Reverted Memory CD8+ T Cells in Patients with X-Linked Lymphoproliferative Disease Caused by Selective Pressure from Epstein-Barr Virus. J. Exp. Med. 209, 913–924. 10.1084/jem.20112391 22493517 PMC3348103

[B41] Panchal N. Booth C. Cannons J. L. Schwartzberg P. L. (2018a). X-Linked Lymphoproliferative Disease Type 1: A Clinical and Molecular Perspective. Front. Immunol. 9, 666. 10.3389/fimmu.2018.00666 29670631 PMC5893764

[B42] Panchal N. Ghosh S. Booth C. (2021). T Cell Gene Therapy to Treat Immunodeficiency. Br. J. Haematol. 192, 433–443. 10.1111/bjh.17070 33280098

[B43] Panchal N. Houghton B. Diez B. Ghosh S. Ricciardelli I. Thrasher A. J. (2018b). Transfer of Gene Corrected T Cells Corrects Humoral and Cytotoxic Defects in X-Linked Lymphoproliferative Disease (XLP1). J. Allergy Clin. Immun. 142, 235–245. 10.1016/j.jaci.2018.02.053 29705247 PMC6034012

[B44] Parolini S. Bottino C. Falco M. Augugliaro R. Giliani S. Franceschini R. (2000). X-Linked Lymphoproliferative Disease. J. Exp. Med. 192, 337–346. 10.1084/jem.192.3.337 10934222 PMC2193227

[B45] Pavel-Dinu M. Wiebking V. Dejene B. T. Srifa W. Mantri S. Nicolas C. E. (2019). Gene Correction for SCID-X1 in Long-Term Hematopoietic Stem Cells. Nat. Commun. 10, 1634. 10.1038/s41467-019-09614-y 30967552 PMC6456568

[B46] Qasim W. Zhan H. Samarasinghe S. Adams S. Amrolia P. Stafford S. (2017). Molecular Remission of Infant B-ALL after Infusion of Universal TALEN Gene-Edited CAR T Cells. Sci. Transl. Med. 9, eaaj2013. 10.1126/scitranslmed.aaj2013 28123068

[B47] Rai R. Romito M. Rivers E. Turchiano G. Blattner G. Vetharoy W. (2020). Targeted Gene Correction of Human Hematopoietic Stem Cells for the Treatment of Wiskott - Aldrich Syndrome. Nat. Commun. 11, 4034. 10.1038/s41467-020-17626-2 32788576 PMC7423939

[B48] Ricciardelli I. Blundell M. P. Brewin J. Thrasher A. Pule M. Amrolia P. J. (2014). Towards Gene Therapy for EBV-Associated Posttransplant Lymphoma with Genetically Modified EBV-Specific Cytotoxic T Cells. Blood 124, 2514–2522. 10.1182/blood-2014-01-553362 25185261 PMC4199953

[B49] Ripamonti A. Provasi E. Lorenzo M. De Simone M. Ranzani V. Vangelisti S. (2017). Repression of miR-31 by BCL6 Stabilizes the Helper Function of Human Follicular Helper T Cells. Proc. Natl. Acad. Sci. U.S.A. 114, 12797–12802. 10.1073/pnas.1705364114 29133396 PMC5715737

[B50] Rivat C. Booth C. Alonso-Ferrero M. Blundell M. Sebire N. J. Thrasher A. J. (2013). SAP Gene Transfer Restores Cellular and Humoral Immune Function in a Murine Model of X-Linked Lymphoproliferative Disease. Blood 121, 1073–1076. 10.1182/blood-2012-07-445858 23223356 PMC3779401

[B51] Rogers G. L. Huang C. Clark R. D. E. Seclén E. Chen H.-Y. Cannon P. M. (2021). Optimization of AAV6 Transduction Enhances Site-Specific Genome Editing of Primary Human Lymphocytes. Mol. Ther. Methods Clin. Dev. 23, 198–209. 10.1016/j.omtm.2021.09.003 34703842 PMC8517001

[B52] Ruffo E. Malacarne V. Larsen S. E. Das R. Patrussi L. Wülfing C. (2016). Inhibition of Diacylglycerol Kinase α Restores Restimulation-Induced Cell Death and Reduces Immunopathology in XLP-1. Sci. Transl. Med. 8, 321ra7. 10.1126/scitranslmed.aad1565 PMC4918505 26764158

[B53] Ryan D. E. Taussig D. Steinfeld I. Phadnis S. M. Lunstad B. D. Singh M. (2018). Improving CRISPR-Cas Specificity with Chemical Modifications in Single-Guide RNAs. Nucleic Acids Res. 46, 792–803. 10.1093/nar/gkx1199 29216382 PMC5778453

[B54] Sayos J. Wu C. Morra M. Wang N. Zhang X. Allen D. (1998). The X-Linked Lymphoproliferative-Disease Gene Product SAP Regulates Signals Induced through the Co-Receptor SLAM. Nature 395, 462–469. 10.1038/26683 9774102

[B55] Sharifi R. Sinclair J. C. Gilmour K. C. Arkwright P. D. Kinnon C. Thrasher A. J. (2004). SAP Mediates Specific Cytotoxic T-Cell Functions in X-Linked Lymphoproliferative Disease. Blood 103, 3821–3827. 10.1182/blood-2003-09-3359 14726378

[B56] Song L. Kauss M. A. Kopin E. Chandra M. Ul-Hasan T. Miller E. (2013). Optimizing the Transduction Efficiency of Capsid-Modified AAV6 Serotype Vectors in Primary Human Hematopoietic Stem Cells *In Vitro* and in a Xenograft Mouse Model *In Vivo* . Cytotherapy 15, 986–998. 10.1016/j.jcyt.2013.04.003 23830234 PMC3711144

[B57] Stadtmauer E. A. Fraietta J. A. Davis M. M. Cohen A. D. Weber K. L. Lancaster E. (2020). CRISPR-Engineered T Cells in Patients with Refractory Cancer. Science 367, eaba7365. 10.1126/science.aba7365 32029687 PMC11249135

[B58] Sweeney C. L. Pavel-Dinu M. Choi U. Brault J. Liu T. Koontz S. (2021). Correction of X-CGD Patient HSPCs by Targeted CYBB cDNA Insertion Using CRISPR/Cas9 with 53BP1 Inhibition for Enhanced Homology-Directed Repair. Gene Ther. 28, 373–390. 10.1038/s41434-021-00251-z 33712802 PMC8232036

[B59] Tebas P. Stein D. Tang W. W. Frank I. Wang S. Q. Lee G. (2014). Gene Editing of CCR5 in Autologous CD4 T Cells of Persons Infected with HIV. N. Engl. J. Med. 370, 901–910. 10.1056/nejmoa1300662 24597865 PMC4084652

[B60] Vakulskas C. A. Behlke M. A. (2019). Evaluation and Reduction of CRISPR Off-Target Cleavage Events. Nucleic Acid Ther. 29, 167–174. 10.1089/nat.2019.0790 31107154 PMC6686686

[B61] Vakulskas C. A. Dever D. P. Rettig G. R. Turk R. Jacobi A. M. Collingwood M. A. (2018). A High-Fidelity Cas9 Mutant Delivered as a Ribonucleoprotein Complex Enables Efficient Gene Editing in Human Hematopoietic Stem and Progenitor Cells. Nat. Med. 24, 1216–1224. 10.1038/s41591-018-0137-0 30082871 PMC6107069

[B62] Villemure J.-F. Savard N. Belmaaza A. (2001). Promoter Suppression in Cultured Mammalian Cells Can Be Blocked by the Chicken β-Globin Chromatin Insulator 5′HS4 and Matrix/Scaffold Attachment Regions. J. Mol. Biol. 312, 963–974. 10.1006/jmbi.2001.5015 11580242

[B63] Wang J. DeClercq J. J. Hayward S. B. Li P. W.-L. Shivak D. A. Gregory P. D. (2015). Highly Efficient Homology-Driven Genome Editing in Human T Cells by Combining Zinc-Finger Nuclease mRNA and AAV6 Donor Delivery. Nucleic Acids Res. 44, e30. 10.1093/nar/gkv1121 26527725 PMC4756813

[B70] Wang M. Sun J. Crosby A. Woodard K. Hirsch M. L. Samulski R. J. Li C. (2017). Direct Interaction of Human Serum Proteins With AAV Virions to Enhance AAV Transduction: Immediate Impact on Clinical Applications. Gene Ther. 24 (1), 49–59. 10.1038/gt.2016.75 27834949 PMC5269444

[B64] Wang X. Chu Y. Wang W. Yuan W. (2016). mTORC Signaling in Hematopoiesis. Int. J. Hematol. 103, 510–518. 10.1007/s12185-016-1944-z 26791377

[B72] Weng X. Liao C. M. Bagchi S. Cardell S. L. Stein P. L. Wang C. R. (2014). The Adaptor Protein SAP Regulates Type II NKT-Cell Development, Cytokine Production, and Cytotoxicity Against Lymphoma. Eur. J. Immunol. 44 (12), 3646–57. 10.1002/eji.201444848 25236978 PMC4261009

[B71] Yang H. Qing K. Keeler G. D. Yin L. Mietzsch M. Ling C. (2020). Enhanced Transduction of Human Hematopoietic Stem Cells by AAV6 Vectors: Implications in Gene Therapy and Genome Editing. Mol. Ther. Nucleic Acids 20, 451–458. 10.1016/j.omtn.2020.03.009 32276210 PMC7150427

[B65] Yang J. Geng L. Ma Y. Tang X. Peng H. Tian J. (2021). SLAMs Negatively Regulate IL-21 Production in Tfh-Like Cells from Allergic Rhinitis Patients. J. Asthma Allergy 14, 361–369. 10.2147/jaa.s291879 33880041 PMC8053523

[B66] Ye J. Coulouris G. Zaretskaya I. Cutcutache I. Rozen S. Madden T. L. (2012). Primer-BLAST: A Tool to Design Target-Specific Primers for Polymerase Chain Reaction. Bmc Bioinformatics 13, 134. 10.1186/1471-2105-13-134 22708584 PMC3412702

[B67] Zetsche B. Gootenberg J. S. Abudayyeh O. O. Slaymaker I. M. Makarova K. S. Essletzbichler P. (2015). Cpf1 Is a Single RNA-Guided Endonuclease of a Class 2 CRISPR-Cas System. Cell 163, 759–771. 10.1016/j.cell.2015.09.038 26422227 PMC4638220

[B68] Zhang L. Zuris J. A. Viswanathan R. Edelstein J. N. Turk R. Thommandru B. (2021). AsCas12a Ultra Nuclease Facilitates the Rapid Generation of Therapeutic Cell Medicines. Nat. Commun. 12, 3908. 10.1038/s41467-021-24017-8 34162850 PMC8222333

