# Supplementary material for: Genome Editing With TALEN, CRISPR-Cas9 and CRISPR-Cas12a in Combination With AAV6 Homology Donor Restores T Cell Function for XLP
Source: Front Genome Ed. 2022 May 23;4:828489. doi: 10.3389/fgeed.2022.828489 (PMC9168036; doi:10.3389/fgeed.2022.828489)
Supplement: Supplementary file 1 [file DataSheet1.PDF]

Supplementary Material

1 Supplementary Data

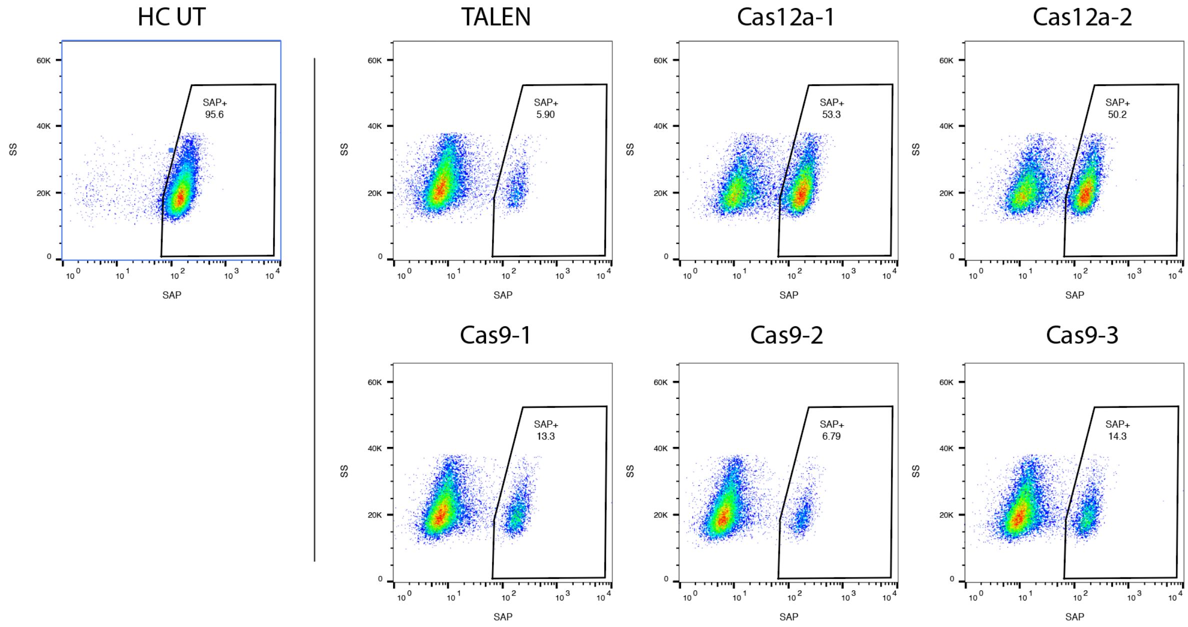

Supplementary figure 1 Intracellular SAP protein staining in healthy control T cells nucleofected with TALEN mRNA, or CRISPR-Cas12a and CRISPR-Cas9 RNPs

Representative flow plots show gated SAP+ population. This number was subtracted from 100 to give the data shown in Figure 1C.

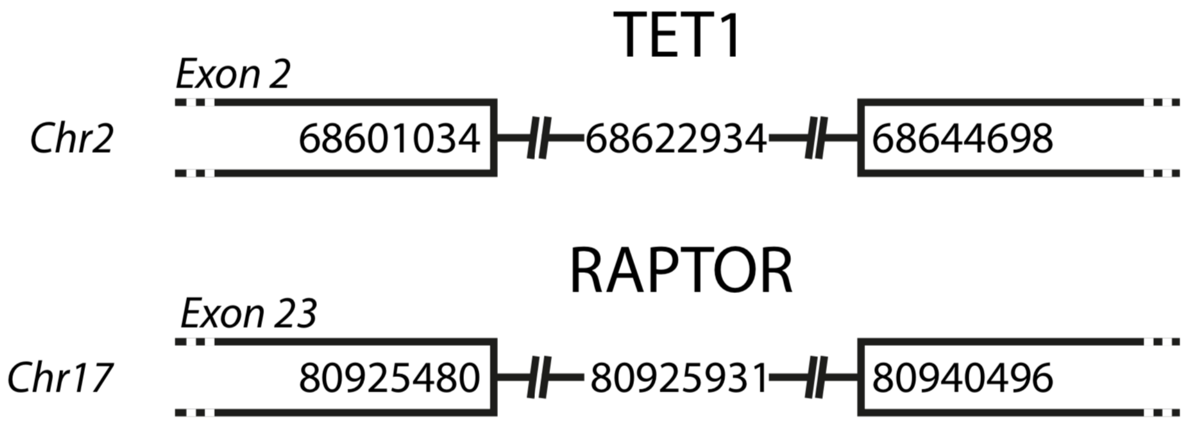

Supplementary figure 2 Off target genomic loci for TALEN and Cas9-3 nucleases

Schematic diagram to show genomic co-ordinates of TALEN OT2 (TET1) and Cas9-3 OT1 (RPTOR).

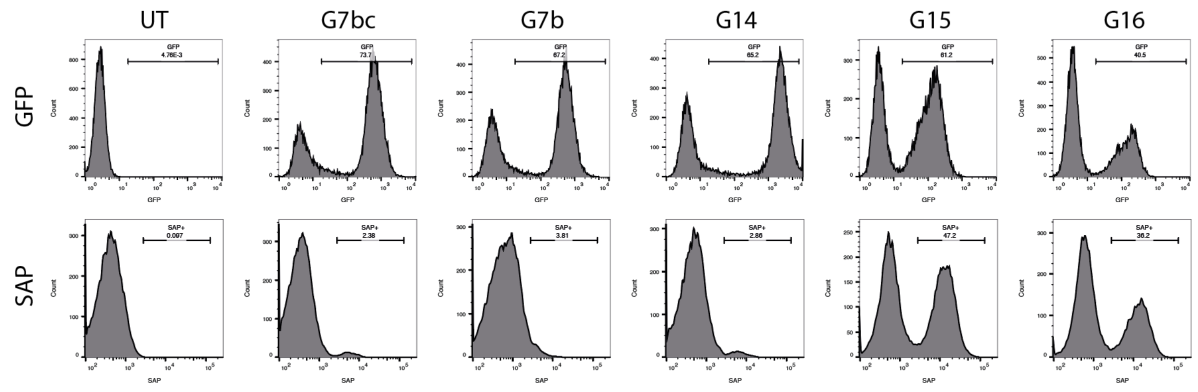

Supplementary figure 3 Flow cytometry of XLP patient cells edited with HDR donors G7bc, G7b, G14, G15 and G15

Histograms obtained through FlowJo analysis of XLP patient T cells edited with all HDR donors. Unstained cells were used for GFP analysis, due to the intracellular SAP staining protocol bleaching GFP fluorescence in stained cells.

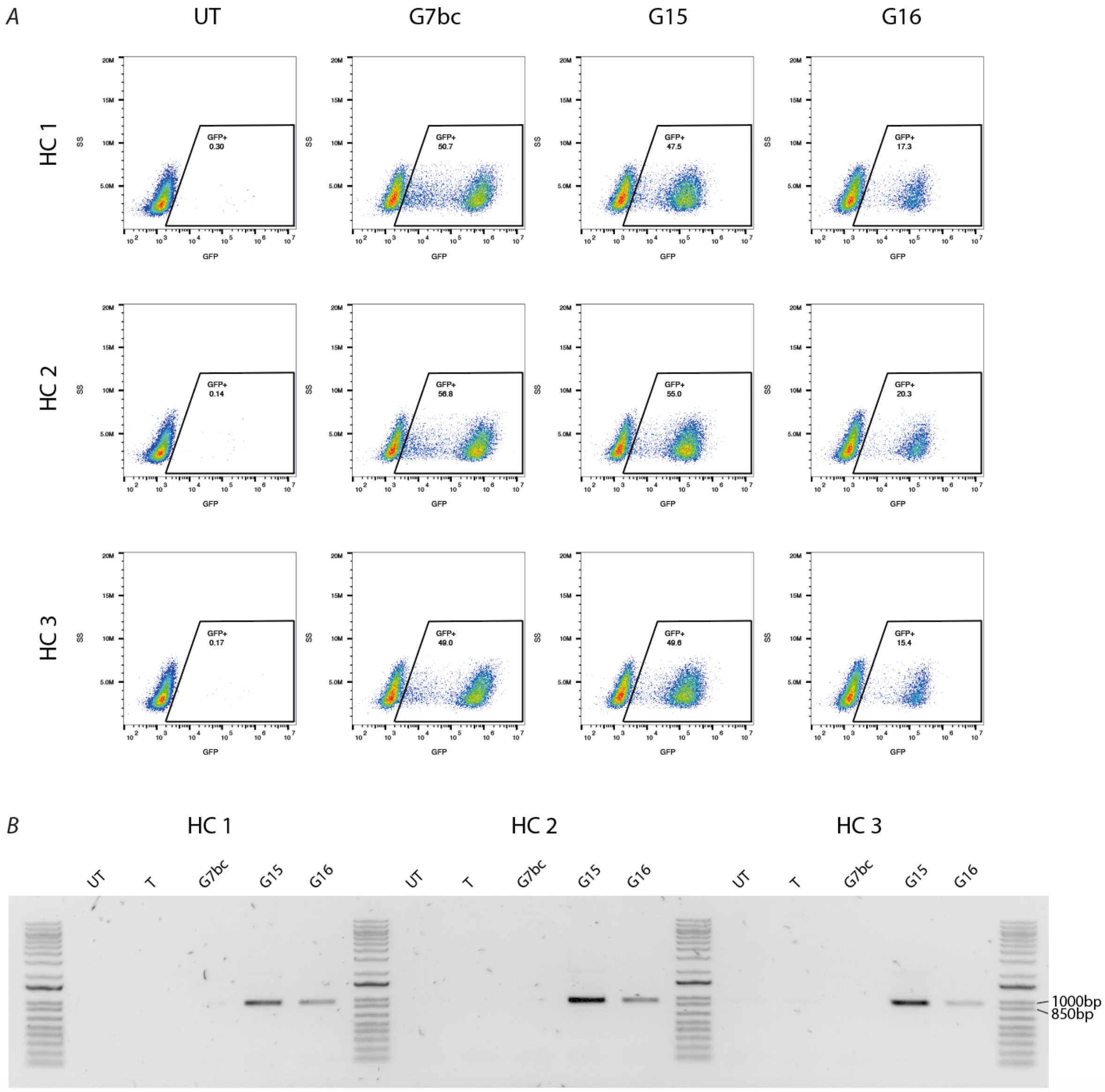

Supplementary figure 4 Flow cytometry dot plots of healthy control T cells edited with HDR donors G7bc, G15 or G16, and in/out PCR performed on the extracted DNA

[A] Flow cytometry dots plots showing the %GFP+ heathy donor T cells after gene editing with G7bc, G15 or G16 HDR donor compared to unedited control (UT). GFP+ determined by flow cytometry at day 3 post edit (n=3, healthy control (HC) 1-3). [B] Gel electrophoresis of an in/out PCR performed using primers specific to the integration of the codon optimized SAP cDNA at the SH2D1A locus, expected band at 953bp. The template DNA was extracted from the samples in [A], after they had undergone FACS-sorting for GFP positive cells. Ladder is 1Kb Plus (Thermo fisher), relevant band sizes indicated.

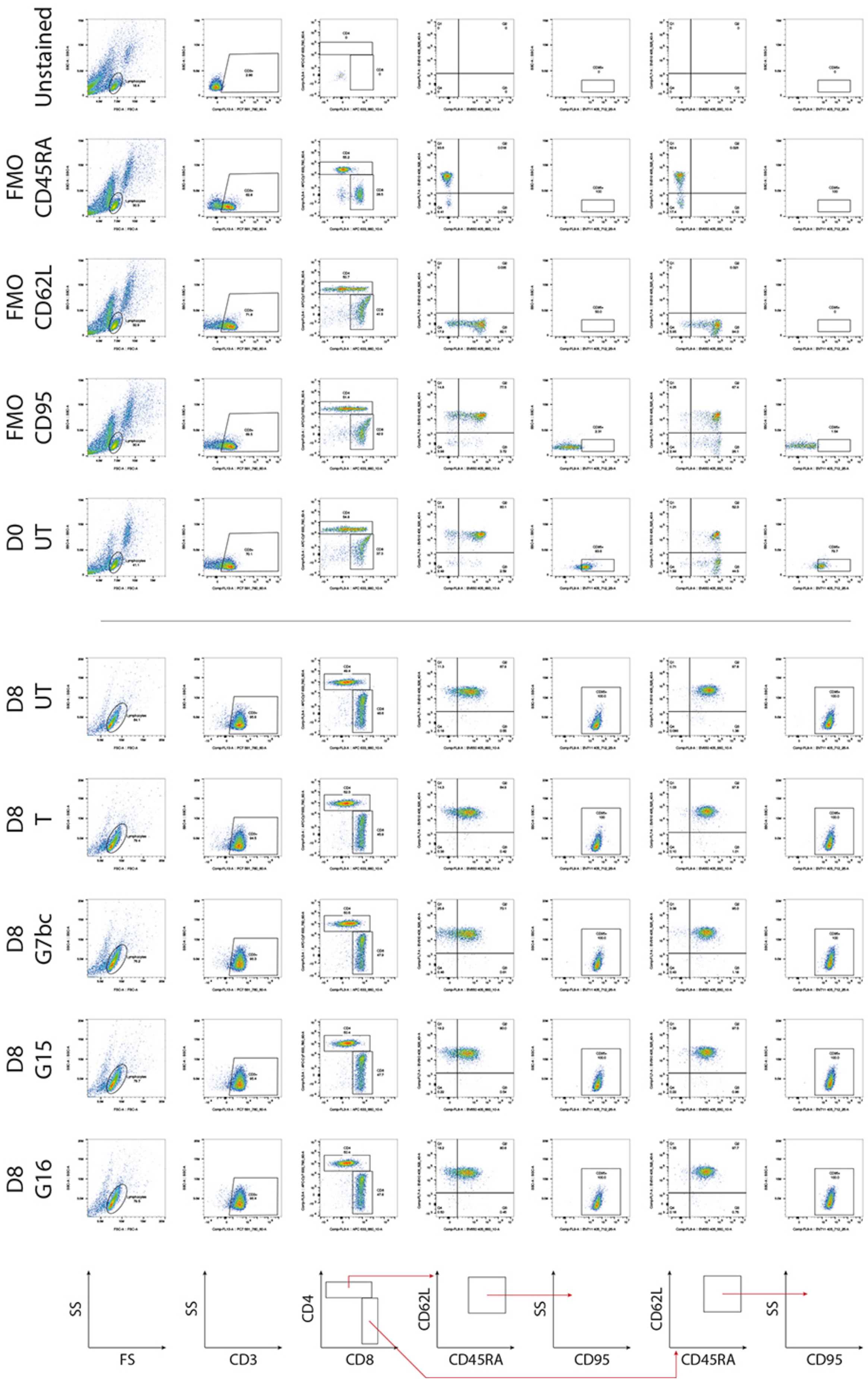
Unstained
FMO CD45RA
FMO CD62L
FMO CD95
D0 UT
D8 UT
D8 T
D8 G7bc
D8 G15
D8 G16
SS
FS
SS
CD3
CD4
CD8
CD62L
CD45RA
SS
CD95
CD62L
CD45RA
SS
CD95

Supplementary figure 5 Representative flow cytometry data of T cell phenotyping flow cytometry panel performed on gene edited healthy control T cells

FACS plots and schematic diagram show representative data and gating strategy used for T cell phenotyping on D0 and D8 (D8=D4 post-edit). Top panel shows unstained (US) and fluorescence minus one (FMO) controls for CD62L, CD45RAand CD95 antibodies to demonstrate how gates were established (these controls were also performed on D8 – data not shown). Bottom panel, representative plots show data post edit, indicating no changes in populations been unedited samples (UT) and those treated with TALENs (T) or HDR donors G7bc, G15 or G16.

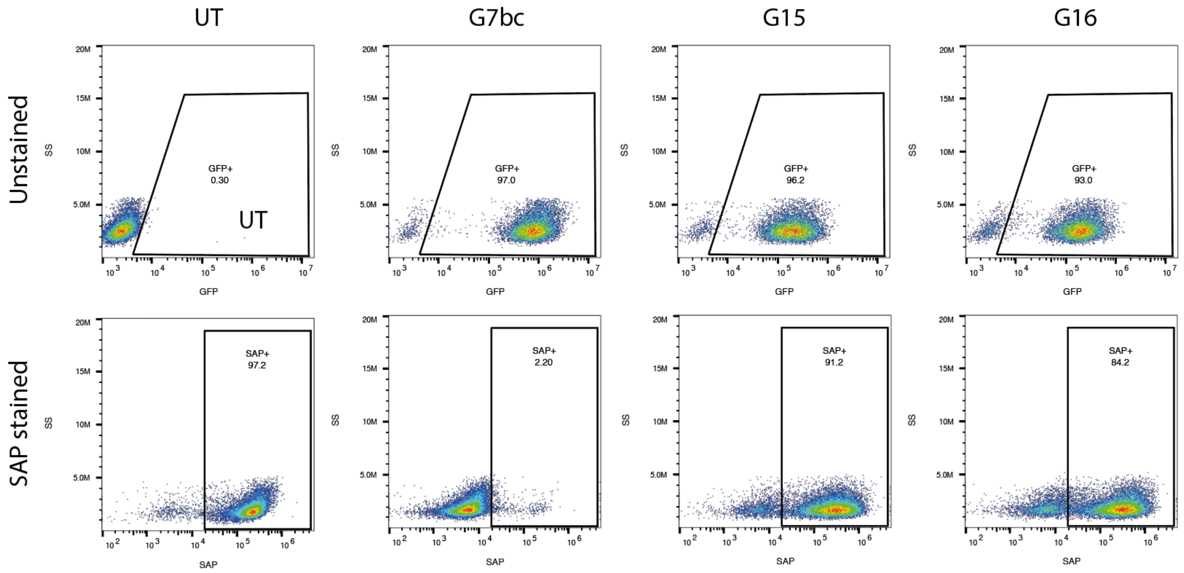

Supplementary figure 6 Representative flow cytometry data of healthy control T cells after FACS-sorting for GFP positive cells

Healthy control T cells edited using TALENs and G7bc, G15 and G16 HDR donors were sorted for GFP positive cells. After this procedure, the percentage of cells GFP+ was analysed using flow cytometry on unstained cells (top row). The same cell populations were stained for SAP expressing via intracellular SAP staining and analysed by flow cytometry (bottom row).

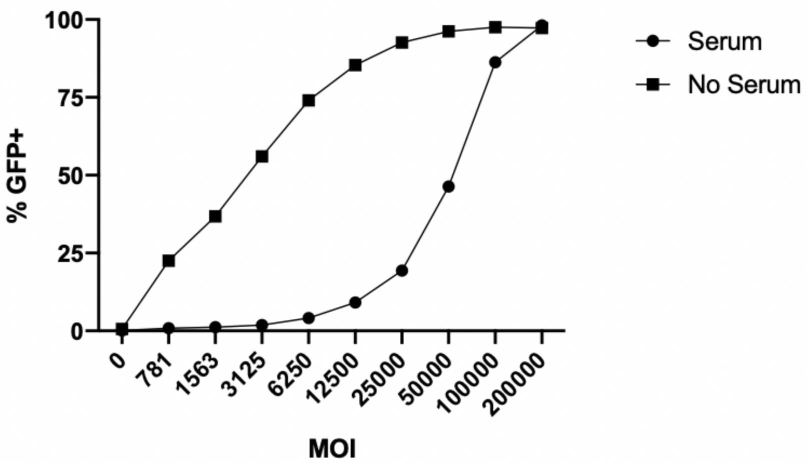

Supplementary figure 7 Effect of FBS on Jurkat transduction with AAV6 HDR donor

[A] %GFP+ Jurkat T cells after transduction with range of G7bc AAV MOIs in the presence or absence of FBS. Cells did not receive electroporation, GFP+ determined by flow cytometry at day 3 (n=1).

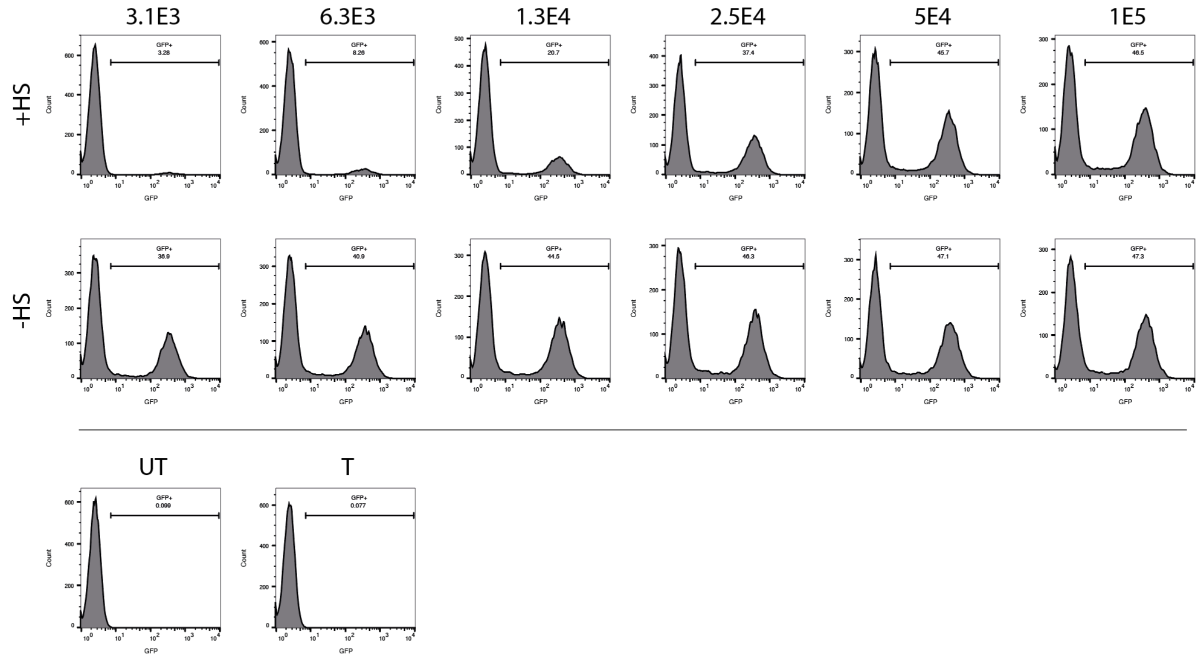

Supplementary figure 8 Effect of human serum (HS) on T cell gene editing

T cells were transduced with G15 donor at a range of doses (3.1E3-1E5) 2 hours prior to TALEN nucleofection. Histograms show representative data acquired by flow cytometry. Gates established based on unedited (UT) and TALEN only (T) controls.

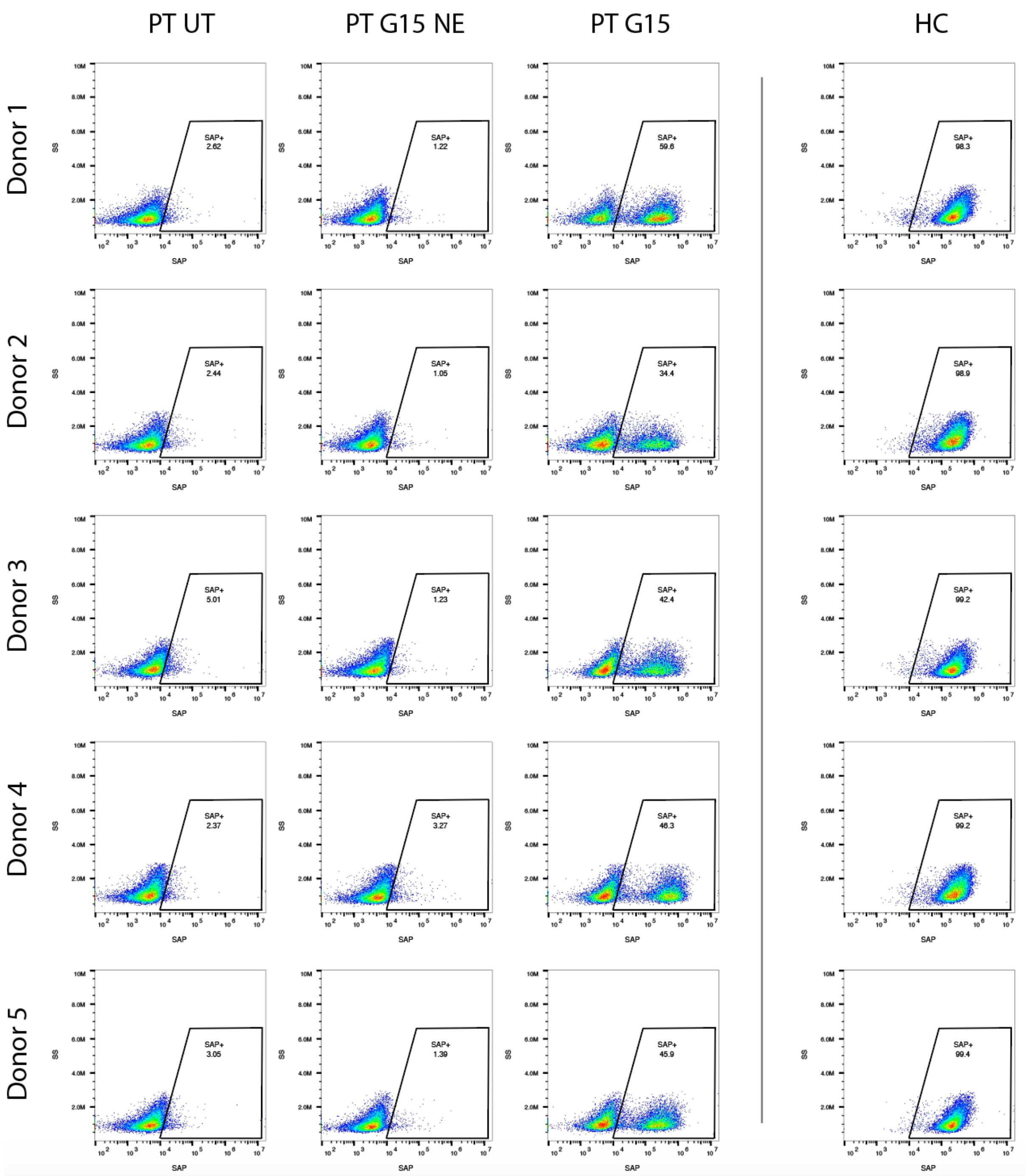

Supplementary figure 9 Flow cytometry dot plots of edited XLP patient T cells Dot plots show SAP expression in XLP patient T cells edited with HDR donor G15 (PT G15), compared to unedited controls (PT UT), G15 AAV-transduced but not electroporated (PT G15 NE) controls, and healthy control cells. Intracellularly SAP staining on d6 post edit (n=5)

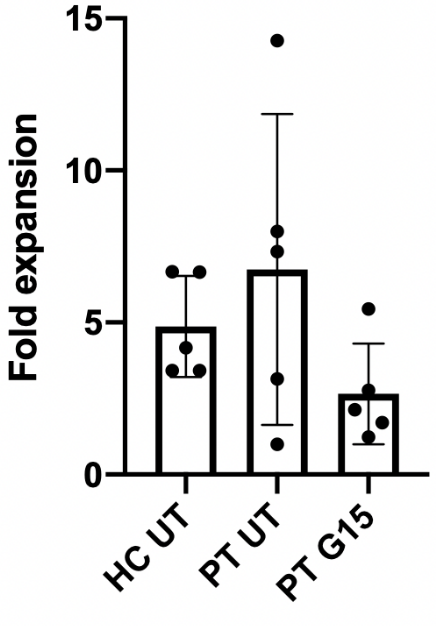

Supplementary figure 10 Fold cell expansion of patient cells editied with TALEN and G15 HDR donor Graph shows the fold expansion of healthy control cells, unedited patient cells, and gene edited XLP patient cells between day 5 (cells put into an editing procedure) and day 9.

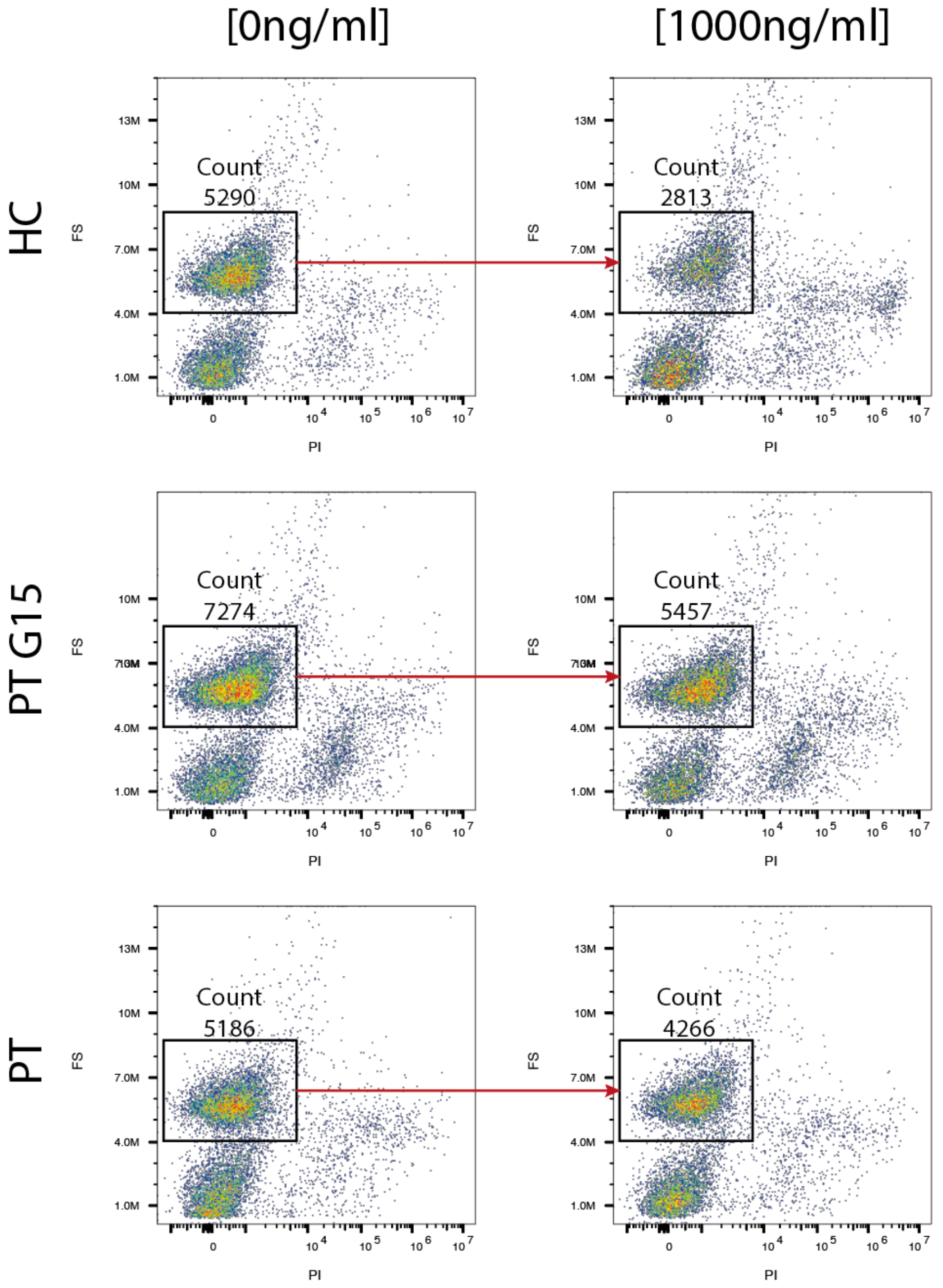

Supplementary figure 11 Representative flow cytometry dot plots of RICD assay

Cell counts in the live gate were obtained from FlowJo software, and then amount of cell loss relative to the 0ng/nl OKT condition was calculated as described in the methods. These representative plots show unedited healthy control cells (HC), XLP patient cells edited with G15 HDR donor (PT G15) and unedited XLP patient T cell control (PT UT), treated with 0ng/ml OKT3 and 1000ng/ml OKT3.

Cas12a-1	Binding sequence	PAM		Location	Gene	Forward primer	Reverse primer
ON	CCATGATACACAGCCACTGCG	TTTG		chrX:-124346651	SH2D1A	CTGGGAGTCAGGTGGTTGAC	CTCGCTGTCCCTCAGCAAAT
OT1	CCATGAAACACTACCACTGCA	TTTC		chr18:+2424130		TATGACAACTTTCCCCTGGTCA	CGAATCTATCCAGGTGCTCAGT
OT2	CCATCAACCACAGCCACTGCA	TTTG		chr2:-213730258		ACCATCAGAGGAATCCCATTTC	GCTGCTCTTATGGTAAAAATATGCT
OT3	CCTTAATACACAGCCATTGTG	TTTG		chr4:-78706036		GGTCTGGGTTTCATTCAAAATGTG	TTAAAGGCTTTGAGGCCAGTCC
OT4	CCATTTTACACAGTCACTGGC	TTTA		chr6:-141264027		CCACTTGAGAAACATTCCGATGA	ATGTTGGATGGGGTAGCAGG
OT5	CCCTGATCCACAGCCCCAGCC	TTTC		chr3:+134726770		CAAGTACTGGTCAATAAAGGGC	CTCCGGCCGGCAGTT
OT6	CCATGGAACACAGCCACATCT	TTTC		chr12:-45062523		AGGTGTTTTACAGATAAGGGTCA	AGGCTATAAGGGAAGATGGTCA
OT7	CAAAGATACATAGCCTCTGCT	TTTC		chr8:-18761247		ACGCAAATCAAACGTCAGGT	GTTTTAAGACTTTTTAGAGGCGAGT
OT8	CCATTATAAACAGCCATGGCT	TTTA		chr1:+6218614		CTGGCTGGGCCTCTTTGTAA	GGGAACAGTCCTCGGTCACT
OT9	CCAGGATACGCTGCCACTTCC	TTTG		chr19:+39160005		ACTGGATCTGTGTCTTTATGGGC	GCTGTTCAGCAAGTAAGCTGG
OT10	CCCTGGAACACAGCCACAGCA	TTTA		chr17:-4721649		GATTCTGTTAGCTTGTGGGGG	ATCTGTGTGCAGGCGAGTTAG
**Cas9-3**	**Binding sequence**	**PAM**		**Location**	**Gene**	**Forward primer**	**Reverse primer**
ON	ATACACAGCCACTGCGTCCA	TGG		chrX:-124346647	SH2D1A	CTGGGAGTCAGGTGGTTGAC	CTCGCTGTCCCTCAGCAAAT

OT1	GAACACAGCCACTGCGTCCA	CAG		chr17:-80925931		GTCTGGGGACTGTTAGACGC	CACTGGGACTGTGCTGACAA
OT2	ATTCACAGCAGCTGCGTCCA	TAG		chr18:+79278188		TTGCAGGTATAGCAAAAGTGGCA	CCATCGAGTTCGAGTGAAGC
OT3	TGGCGCAGCCACTGCGTCCA	GGG		chr19:-1762160		GACCCCCATCCGCCC	CAGCTTCTGTCCCGACTCC
OT4	CAACACAGCCACTGTGTCCA	GGG		chr17:+1156951		ATGGTGAGGCTGGGATCTGA	GTGAGGCTGCGGTGAGTTA
OT5	ATACACACCTACTGTGTCCA	AGG		chr1:+22127989		TGTTTGCGGTGGAGGACATT	CAACAGAGACCCAGTGAGCA
OT6	TTGCACAGCCACTGCCTCCA	AGG		chr10:+78377254		GGTGCTCATTGGCCACAGT	GAAAGCAAGAGCTCCCCTCA
OT7	AAAACCAGCCACCGCGTCCA	TAG		chr7:-98017877		TCAGGCTGAGAAGGAACACG	AAAAAGTCCCCCAAGGGCAG
OT8	CTCCACAGCCGCTGCGTCCC	GGG		chr13:+36214593	SOHLH2	CAGTGCTCCTGGCAGATAATTGAG	CCCGGGTTCCGCGTTGA
-	CTCCCCAGCCACTGTGTCCA	TGG		chr12:-117110314		Not identified	Not identified
OT9	CTTGACAGCCACTGTGTCCA	AGG		chr16:-13340167		CTGATTTAAGATGTGCTCAACCTTC	GGGAAAGACACCTGGCCAAATA
OT10	AACCACAGCCAGTGCGTCCC	TGG		chr2:-25378890		GGCTTGGTCAGCCCTGTAGTC	GCATTGGCAGACCCGACA
**TALEN**	**Left half site**	**Right half site**	**Orientation**	**Location**	**Gene**	**Forward primer**	**Reverse primer**
ON	TGCCCAAGAGTCCACCAGG	GGCTGTGTATCATGGCAAA	L-13-R	chrX:124346622	SH2D1A	CTGTTGTTGGGGTGCTTCTCTC	CTCACAGCACACATAGGCAGTAC
OT1	TTTGCCtTaATACACCcaCC	atCctTtTATCATGGtAAA	R-28-R	chr13:74015878		CCCTTTCTTCCCAAGGTAGGTC	GAGACATTTGCTAGGGAGGATGAG

OT2	TTgGCaATaATcCACAGga	tGCTGTGTATCATGtCAgA	R-11-R	chr10:68622934		AAAGGACCCTCCCGCCTTAG	CATAGTCTCCAAGTATCTCCCCAC
OT3	TTTGCCAaaAgACACAtaC	tGtTGTtTATCATaGtAAA	R-14-R	chr5:56408412		GCACCTTGCAGAAAGCATTTTCCC	CCTGACATCTAACACTGTAGATTAGTTTTGCC
OT4	TTcaCCATtATACACAtCC	GGCcGgGcAcCATGGCttA	R-23-R	chr3:136206633		CCCTCCTTTATCTTCCTCCACC	GCACCAGCACACCCAGCTAA
OT5	TTTtCCATGAgACACAcCC	GtCaGTtTATCATtGCcAt	R-13-R	chr3:86273842		CTTCTCTTGTGTCTTGAACTACATCAACAATAGG	CAAAGGCAGTTCTGCAGTCACATG
OT6	TTTtCCtTtATACACAaCC	GcaTaTaTATCtTGGCAAA	R-17-R	chr16:87976124		GTGTGTGGTGTCATGGAACCTTAC	GTTCTCAAGCACTGTCCGTGG
OT7	TGCCCAgGAcagaACCtGG	CCTGGTGGcCTCTTGGGCc	L-20-L	chr1:23792545		ACACATGTTCTGGGCCACTAGG	GGGGCACAGACATGGTGTTAC
OT8	TTTGCCATGAagCAaAGCC	GGCTGTGaATgAaGGggAg	R-15-R	chr22:25845903		GGTGTCGTAGCATTGCTGTAGG	TAGGAGGTTCTTGGGAAAGCCTTG
OT9	TcaGCCATGgTgCcCAGCt	CCTGGTGtACTCcTtGGtA	R-19-L	chr18:12832398		CGCCACCACACCTGGCTAAA	GAGTGAGGAAAACGCCACTCTCAA
OT10	aTTGCCtTtATtCAaAtCC	GGCTaTGTATCtaGGCAAA	R-17-R	chr10:16900359		CTCAGCATTCTGCTTTTAGGCTCC	GGTGGTGGATTTTATGCTACGTGG

Supplementary Table 1. Primer sequences for Cas12a-1, Cas9-3 and TALEN on and off target PCR amplicons. Primers are given in the 5'-3' orientation. For Cas12a-1, Cas9-3, the off-target positions are highlighted in red, TALEN mismatches are denoted by a lowercase letter. For TALEN orientation, the letter denotes Left (L) or right (R) TALEN arm, the number indicating the spacer difference. TALEN off targets were identified in silico by PROGNOS, Cas9-3 and Cas12a-1 by Benchling.

	UT	T	T+G7bc	T+G15	T+G16
HC1	0.00%	0.00%	0.00%	84.84%	80.67%
HC2	0.01%	0.02%	0.02%	91.01%	82.40%
HC3	0.54%	0.85%	0.00%	85.28%	73.85%

Supplementary table 2 ddPCR-derived editing frequencies of FACS-sorted healthy control T cells
